# *SCP2* variant is associated with alterations in lipid metabolism, brainstem neurodegeneration, and testicular defects

**DOI:** 10.1186/s40246-022-00408-w

**Published:** 2022-08-22

**Authors:** Melanie Galano, Shereen Ezzat, Vassilios Papadopoulos

**Affiliations:** 1 Department of Pharmacology and Pharmaceutical Sciences, School of Pharmacy, University of Southern California, 1985 Zonal Ave, Los Angeles, CA 90089 USA; 2 Department of Medicine, University of Toronto and Princess Margaret Cancer Center, Toronto, ON M5G 2C1 Canada

**Keywords:** Lipid transfer proteins, Lipids/oxidation, Peroxisomes, Cholesterol, Fatty acid

## Abstract

**Background:**

The detoxification of very long-chain and branched-chain fatty acids and the metabolism of cholesterol to form bile acids occur largely through a process called peroxisomal β-oxidation. Mutations in several peroxisomal proteins involved in β-oxidation have been reported, resulting in diseases characterized by neurological defects. The final step of the peroxisomal β-oxidation pathway is catalyzed by sterol carrier protein-x (SCPx), which is encoded by the *SCP2* gene. Previously, there have been two reports of SCPx deficiency, which resulted from a homozygous or compound heterozygous *SCP2* mutation. We report herein the first patient with a heterozygous *SCP2* mutation leading to SCPx deficiency.

**Results:**

Clinical presentations of the patient included progressive brainstem neurodegeneration, cardiac dysrhythmia, muscle wasting, and azoospermia. Plasma fatty acid analysis revealed abnormal values of medium-, long-, and very long-chain fatty acids. Protein expression of SCPx and other enzymes involved in β-oxidation were altered between patient and normal fibroblasts. RNA sequencing and lipidomic analyses identified metabolic pathways that were altered between patient and normal fibroblasts including PPAR signaling, serotonergic signaling, steroid biosynthesis, and fatty acid degradation. Treatment with fenofibrate or 4-hydroxytamoxifen increased SCPx levels, and certain fatty acid levels in patient fibroblasts.

**Conclusions:**

These findings suggest that the patient’s *SCP2* mutation resulted in decreased protein levels of SCPx, which may be associated with many metabolic pathways. Increasing SCPx levels through pharmacological interventions may reverse some effects of SCPx deficiency. Collectively, this work provides insight into many of the clinical consequences of SCPx deficiency and provides evidence for potential treatment strategies.

**Supplementary Information:**

The online version contains supplementary material available at 10.1186/s40246-022-00408-w.

## Background

Mitochondria and peroxisomes are the two sites of fatty acid β-oxidation, with the metabolism of short-, medium-, and long-chain fatty acids occurring in mitochondria and the chain shortening of very long-chain fatty acids (VLCFA), long-chain dicarboxylic acids, 2-methyl-branched fatty acyl-CoAs, eicosanoids, and bile acid precursors taking place in peroxisomes [1]. Peroxisomal β-oxidation of straight-chain fatty acyl-CoAs occurs through the function of acyl-CoA oxidase 1 (ACOX1), L-bifunctional protein (LBP), and 3-ketoacyl-CoA thiolase (ACAA1), while the oxidation of 2-methyl-branched fatty acyl-CoAs is catalyzed by acyl-CoA oxidase 2 (ACOX2), D-bifunctional protein (DBP), and sterol carrier protein-x (SCPx) [1]. In addition to these enzymes, other proteins involved in peroxisomal β-oxidation include ABCD1, which aids importation of straight chain VLCFA into peroxisomes, and 2-methylacyl-CoA racemace (AMACR), which converts fatty acids with a methyl group in the (*R*)-configuration to the (*S*)-configuration to become substrates for β-oxidation [2].

Human diseases caused by a defect in one of these peroxisomal proteins are called single peroxisomal enzyme deficiencies (PEDs). Patients with PED typically present with severe neurological symptoms. The most common PED is called X-linked adrenoleukodystrophy (X-ALD [MIM: 300371]), which is caused by mutations in the *ABCD1* gene, resulting in elevated levels of VLCFA, and most frequently presents with severe cerebral ALD or adrenomyeloneuropathy (AMN) [2]. Other PEDs include ACOX1 deficiency [MIM: 264470], caused by mutations in the *ACOX1* gene and reported in about 30 patients; DBP deficiency [MIM: 261515], caused by mutations in the *HSD17B4* gene encoding DBP and reported in over 100 patients; and AMACR deficiency [MIM: 614307] caused by mutations in the *AMACR* gene and reported in about 10 patients [2]. Lastly, PED can be caused by mutations in the *SCP2* gene resulting in SCPx deficiency [MIM: 613724], which has previously been reported in only two patients. The first patient reported with SCPx deficiency had a homozygous 1-nucleotide insertion (c.545_546insA) and presented with leukoencephalopathy with motor and peripheral neuropathy, dystonia, hyposmia, nystagmus, and azoospermia [3, 4]. The second patient with SCPx deficiency was a compound heterozygote (c.349C > T and c.121G > T) with spinocerebellar ataxia and brain iron accumulation [4].

The *SCP2* gene contains two transcription start sites, resulting in a 58 kDa SCPx and a 15 kDa pro-sterol carrier 2 protein (*SCP2*) [5]. SCPx is post-translationally cleaved in two, resulting in a 45 kDa thiolase, referred to hereafter as SCPx, including its N-terminus and the remainder with its C-terminus resulting in a 13 kDa mature *SCP2* protein. Pro-*SCP2* is also processed to form 13 kDa *SCP2*. Both the 45 kDa SCPx and 13 kDa *SCP2* have been implicated in the transport and metabolism of various lipids including sterols [6–8], fatty acids [9–11], phospholipids [8, 12], and fatty acyl-CoAs [13, 14]. SCPx and *SCP2* are most highly expressed in tissues involved in the oxidation and trafficking of cholesterol: adrenals, ovary, testis, liver, and intestine [15]. While SCPx is almost exclusively localized to peroxisomes, about 50% of *SCP2* is peroxisomal and about 50% is extraperoxisomal (mitochondria, endoplasmic reticulum, and cytosol) [15, 16]. The 45 kDa SCPx exhibits lipid-transfer and sterol-carrier activities and is a 3-ketoacyl-CoA thiolase enzyme that has been shown to be involved in peroxisomal oxidation of branched chain fatty acids, straight chain fatty acids, and the branched side chain of cholesterol [15].

Here, we report a third patient with SCPx deficiency and the first resulting from a missense heterozygous mutation in *SCP2*. The patient is a 59-year-old male presenting with brainstem neurodegeneration and azoospermia. Whole-genome sequencing revealed a c.572A > G heterozygous mutation resulting in a His191Arg substitution. Patient plasma fatty acid analysis revealed abnormalities in the levels of several fatty acids, including γ- and α-linoleic acid and arachidonic acid. Primary dermal fibroblasts from the patient and control normal human dermal fibroblasts (NHDF) were used to investigate a link between the patient’s *SCP2* mutation and its clinical consequences. Western blot analysis revealed a significant reduction in the patient’s SCPx levels compared to NHDF as well as alterations in the levels of many other proteins involved in peroxisomal and mitochondrial β-oxidation. RNA sequencing identified several lipid-related genes that were differentially expressed between patient and normal fibroblasts. Lipidomic analysis identified alterations in several free fatty acids, acylcarnitines, sterols, phospholipids, and sphingolipids involved in metabolic pathways such as steroid and primary bile acid biosynthesis, linoleic metabolism, and fatty acid degradation. Finally, we identified two compounds that have shown to increase SCPx levels and certain fatty acid levels in patient fibroblasts compared to untreated cells. Collectively, our data suggest that the patient’s novel heterozygous *SCP2* mutation is associated with alterations in a significant number of genes and lipids that are involved in several critical lipid metabolic pathways, which may contribute to neurodegeneration and testicular defects as seen in the patient.

## Results

### Identification of a novel heterozygous SCP2 variant

The patient was a 59-year-old Greek Canadian male with a nine-year history of progressive degenerative neurological disease. This included loss of cranial nerve function with progressive inability to swallow, chew, speak, breathe, or control head movement. Craniospinal imaging studies were negative, and the patient showed no cerebellar problems clinically or radiographically. Taken together, these neurological phenotypes are consistent with brainstem, rather than cerebellar, neurodegeneration. In addition to these neurological defects, the patient also had a history of cardiac dysrhythmia without associated risk factors, and primary hypogonadism with azoospermia and reduced testosterone with elevated follicle-stimulating hormone levels.

Whole-genome sequencing identified a novel c.572A > G heterozygous variant resulting in a p.His191Arg substitution. Table 1 summarizes the results of the patient’s genome-wide scan, including other gene variants identified. The p.His191Arg substitution was not previously identified in the literature nor in ClinVar or LOVD 3.0 databases. The variant was identified in dbSNP (rs372168791) and in control databases in 11 of 282,548 chromosomes at a frequency of 0.00003893 (Genome Aggregation Database March 6, 2019, v2.1.1). The variant was observed in European (non-Finnish) in 9 of 129,022 chromosomes (freq: 0.00007) and “Other” in 2 of 7204 chromosomes (freq: 0.000278) populations, but not observed in African, Latino, Ashkenazi Jewish, East Asian, European (Finnish), or South Asian populations. The p.His191 residue is conserved in mammals and other organisms, and 8 of 8 computational analyses (SIFT, FAHTMM, DANN, MT, MetaLR, Revel, PolyPhen, and MutationTaster) suggest that the variant may impact the protein; however, this information is not predictive enough to assume pathogenicity. The variant occurs outside of the splicing consensus sequence, and in silico or computational prediction software programs (SpliceSiteFinder, MaxEntScan, NNSPLICE, and GeneSplicer) do not predict a difference in splicing. In summary, the clinical significance of this variant could not be determined with certainty and was classified as a variant of uncertain significance from the database search, thus necessitating further work to elucidate the clinical significance of this variant.

**Table 1:** Summary of genetic variants identified in the patient by genome-wide scan

Disease (Inheritance)	Gene (Transcript)	Variant; Zygosity	Variant interpretation	Variant frequency (GnomAD)
Osteosclerotic metaphyseal dysplasia (AR); Parkinson’s disease (preliminary evidence, inheritance unknown)	*LRRK1* (NM_024652.6)	c.4909A > T; Heterozygous	Variant of uncertain significance	0
Beta-mannosidosis (AR)	*MANBA* (NM_005908.3)	c.1622G > A; Heterozygous	Likely pathogenic	0.000008
Leukoencephalopathy with dystonia and motor neuropathy (AR)	*SCP2* (NM_002979.5)	c.572A > G; Heterozygous	Variant of uncertain significance	0.00004
Dent disease (XLR); Hypophosphatemic rickets (XLR); Nephrolithiasis, type I (XLR); Proteinuria, low molecular weight, with hypercalciuric nephrocalcinosis (XLR)	*CLCN5* (NM_001127898.4)	c.152G > A, Heterozygous	Variant of uncertain significance	0.00001265 total; 0.00001685 in females; 0 in males
Hypokalemic periodic paralysis, type 1 (AD); Susceptibility to malignant hyperthermia (AD); Susceptibility to thyrotoxic periodic paralysis (AD)	*CACNA1S* (NM_000069.3)	c.262A > G, Heterozygous	Variant of uncertain significance	0.0002369

*AR* Autosomal recessive; *AD* autosomal dominant; *XLR* X-linked recessive

Because of the known role of SCPx in fatty acid metabolism, the patient’s plasma fatty acid levels were measured after an overnight fast on two separate occasions. The resulting fatty acid profile (Table 2) shows that many medium-, long-, and very long-chain fatty acids were outside the respective reference range. Reference ranges were derived from the central 95% of data derived from a large group of healthy individuals established by the clinical biochemistry laboratory. Specifically, the patient’s levels of medium-chain fatty acids octanoic acid (8:0) and decenoic acid (10:0) and long-chain fatty acids hexadecadienoic acid (16:2), g-linolenic acid (18:3w6), a-linolenic acid (18:3w3), and arachidic acid (20:0) were below normal. In contrast, the patient’s levels of long-chain fatty acids arachidonic acid (20:4w6) and mead acid (20:3w9) and VLCFA DHA (22:6w3) and nervonic acid (24:1w9) were above normal (Table 2). These abnormalities in the levels of medium-, long-, and very long-chain fatty acids are consistent with the role of SCPx in fatty acid β-oxidation and suggest that both mitochondrial and peroxisomal fatty acid β-oxidation were affected.

**Table 2:** Patient plasma fatty acid panel

Fatty acid	Patient value (nmol/mL)	Reference value (nmol/mL)
**8:0 (Octanoic acid)**	**5**	**8–47**
**10:1 (Decenoic acid)**	**1.6**	**1.8–5.0**
10:0 (Decanoic acid)	8	2–18
12:1 (Lauroleic acid)	2.2	1.4–6.6
12:0 (Lauric acid)	32	6–90
14:2 (Tetradecadlenoic acid)	1.0	0.8–5.0
14:1 (Myristoleic acid)	5	3–64
14:0 (Myristic acid)	77	30–150
**16:2 (Hexadecadienoic acid)**	**8**	**10–48**
16:1w9 (Hexadecenoic acid)	56	25–105
16:1w7 (Palmitoleic acid)	171	110–1130
16:0 (Palmitic acid)	2307	1480–3730
**18:3w9 (g-Linoleic acid)**	**14**	**16–150**
**18:3w3 (a-Linoleic acid)**	**40**	**50–130**
18:2w6 (Linoleic acid)	2854	2270–3850
18:1w9 (Oleic acid)	1893	650–3500
18:1w7 (Vaccenic acid)	341	280–740
18:0 (Stearic acid)	712	590–1170
20:5w3 (EPA)	29	14–100
**20:4w6 (Arachidonic acid)**	**1555**	**520–1490**
**20:3w9 (Mead acid)**	**87**	**7–30**
20:3w6 (h-g-Linolenic acid)	166	50–250
**20:0 (Arachidic acid)**	**42**	**50–90**
**22:6w3 (DHA)**	**318**	**30–250**
22:5w6 (DPA)	43	10–70
22:5w3 (DPA)	125	20–210
22:4w6 (DTA)	77	10–80
22:1 (Docosenoic acid)	10	4–13
22:0 (Docosanoic acid)	50.8	0.0–96.3
**24:1w9 (Nervonic acid)**	**119**	**60–100**
24:0 (Tetracosanoic acid)	40.8	0.0–91.4
26:1 (Hexacosanoic acid)	0.7	0.3–0.7
26:0 (Hexacosanoic acid)	0.48	0.00–1.30
15:0(CH3)4 (Pristanic acid)	0.09	0.00–2.98
16:0(CH3)4 (Phytanic acid)	0.88	0.00–9.88
Total Fatty Acids	11.2 mmol/L	7.3–16.8 mmol/L

Fatty acids in bold indicate fatty acids out of the reference range

### Characterization of SCPx and SCP2 in NHDF and WESP cells

**Fig. 1:**
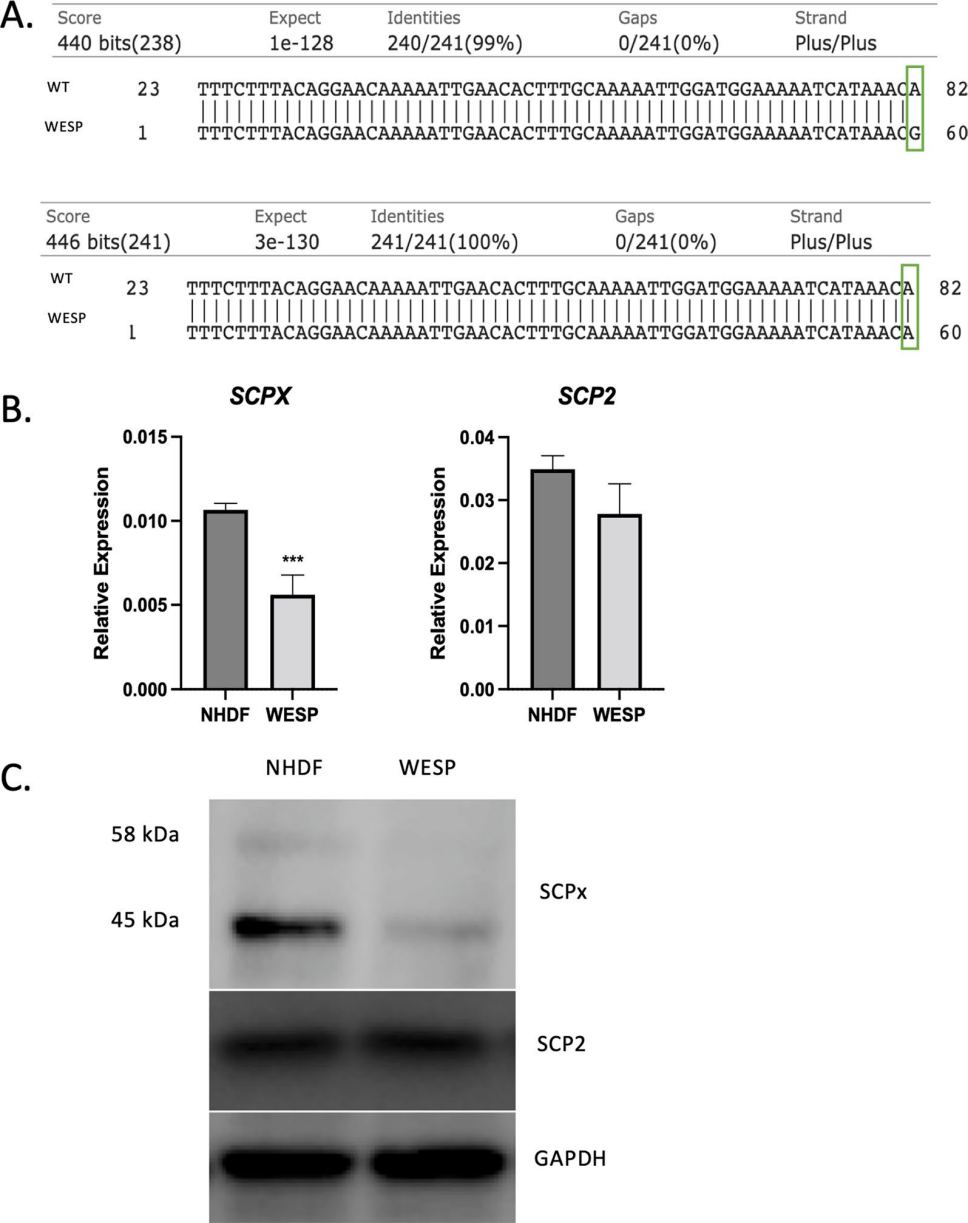
Characterization of *SCP2* mutation in patient fibroblasts. **A** BLAST DNA sequence of amplicon-based NGS results of *SCP2* in patient fibroblasts (WESP) compared to the human wild-type (WT) sequence. Green box shows A > G mutation present in half the reads (top) and WT nucleotide in half the reads (bottom). **B** qRT-PCR analyses of the SCPx coding region of *SCP2* (left) and the SCP2 coding region of *SCP2* (right), where data are normalized to *GAPDH*. Data are shown as mean ± SEM (*n* = 3). ***p < 0.001. **C** Western blot analyses of SCPx and SCP2 in control fibroblasts (NHDF) and WESP cells

Primary fibroblasts (WESP) were generated from the patient, and the heterozygous c.572A > G variant identified by whole-genome sequencing was confirmed by next-generation sequencing (NGS)-based amplicon sequencing. NGS and Basic Local Alignment Search Tool (BLAST) results from WESP cells revealed that about 50% of the reads contained the WT nucleotide, while about 50% of the reads had the c.572A > G mutation, confirming the heterozygous variant in *SCP2* identified via whole-genome sequencing (Fig. 1A). Normal human dermal fibroblasts (NHDF) confirmed as WT for *SCP2* by sequencing were used as controls to compare levels of SCPx in WESP cells. Quantitative real-time polymerase chain reaction (qRT-PCR) was used to measure expression of the SCPx-coding region of the *SCP2* gene (shown as *SCPX*) and showed that there was about 50% lower mRNA expression of this region of *SCP2* in WESP cells compared to controls, consistent with a heterozygous mutation (Fig. 1B). There was no difference in the expression of the *SCP2*-coding region of the *SCP2* gene between the two samples since this region has its own promoter (Fig. 1B). Protein levels of SCPx were examined by immunoblot, which showed that there was a significant reduction in the amount of 58 kDa and 45 kDa SCPx in WESP cells compared to NHDF (Fig. 1C). Consistent with qRT-PCR results, there was no difference in levels of 13 kDa *SCP2* between the two groups (Fig. 1C). These data validate the whole-genome sequencing results and confirm the presence of a heterozygous variant in the SCPx-coding region of the *SCP2* gene, showing that the variant leads to a significant decrease in mRNA expression and protein levels.

### SCPx deficiency is associated with alterations in peroxisome number

**Fig. 2:**
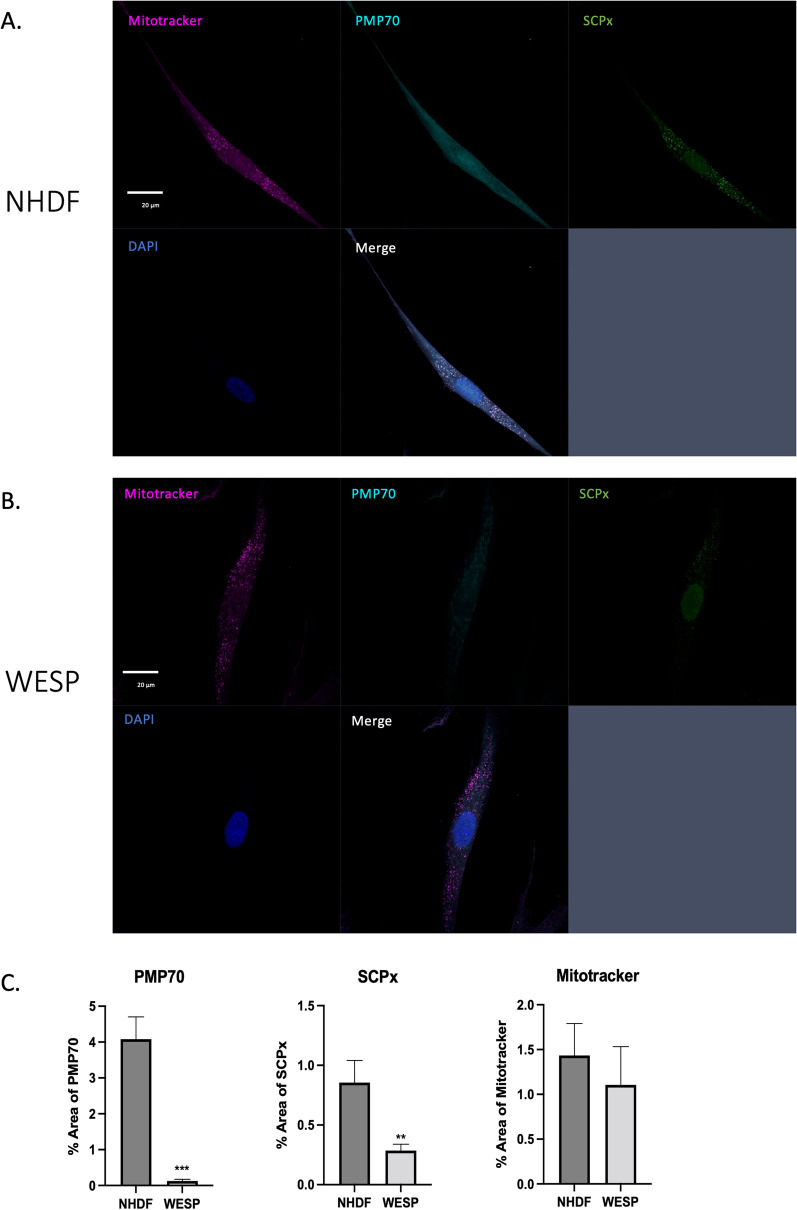
Quantification of peroxisome abundance in NHDF and WESP cells. **A** Confocal microscopy image of NHDF stained with Mitotracker, PMP70 (peroxisomes), SCPx, and DAPI. Scale bar: 20 µm. **B** Confocal microscopy images of a WESP fibroblast stained with Mitotracker, PMP70 (peroxisomes), SCPx, and DAPI. Scale bar: 20 µm. **C** Quantification of confocal images. The area of staining with each marker was quantified in 10 images per sample using ImageJ. Data are shown as mean ± SEM (*n* = 10). **p < 0.01; ***p < 0.001

Next, we were interested in identifying potential changes in the abundance of peroxisomes. Alterations in peroxisome abundance have been reported in fibroblasts of patients with other peroxisomal disorders such as Zellweger’s disease [MIM: 214100] [17]. To determine any changes in the number of peroxisomes in patient WESP cells, we performed immunocytochemistry using the peroxisomal membrane protein 70 (PMP70), a commonly used peroxisomal marker. We found that patient WESP cells had significantly less peroxisomes than control cells (Fig. 2A–C). As a control, we stained for mitochondria using Mitotracker and found that there was no difference in the number of mitochondria between the two cell types (Fig. 2A–C). We also stained for SCPx, which had significantly decreased levels in patient cells compared to control, consistent with the data in Fig. 1 (Fig. 2A–C). These data show that a decrease in SCPx levels is associated with a decrease in the number of peroxisomes, which is consistent with other peroxisomal disorders associated with neurodegenerative phenotypes.

### Alterations in levels of other peroxisomal and mitochondrial β-oxidation enzymes

**Fig. 3:**
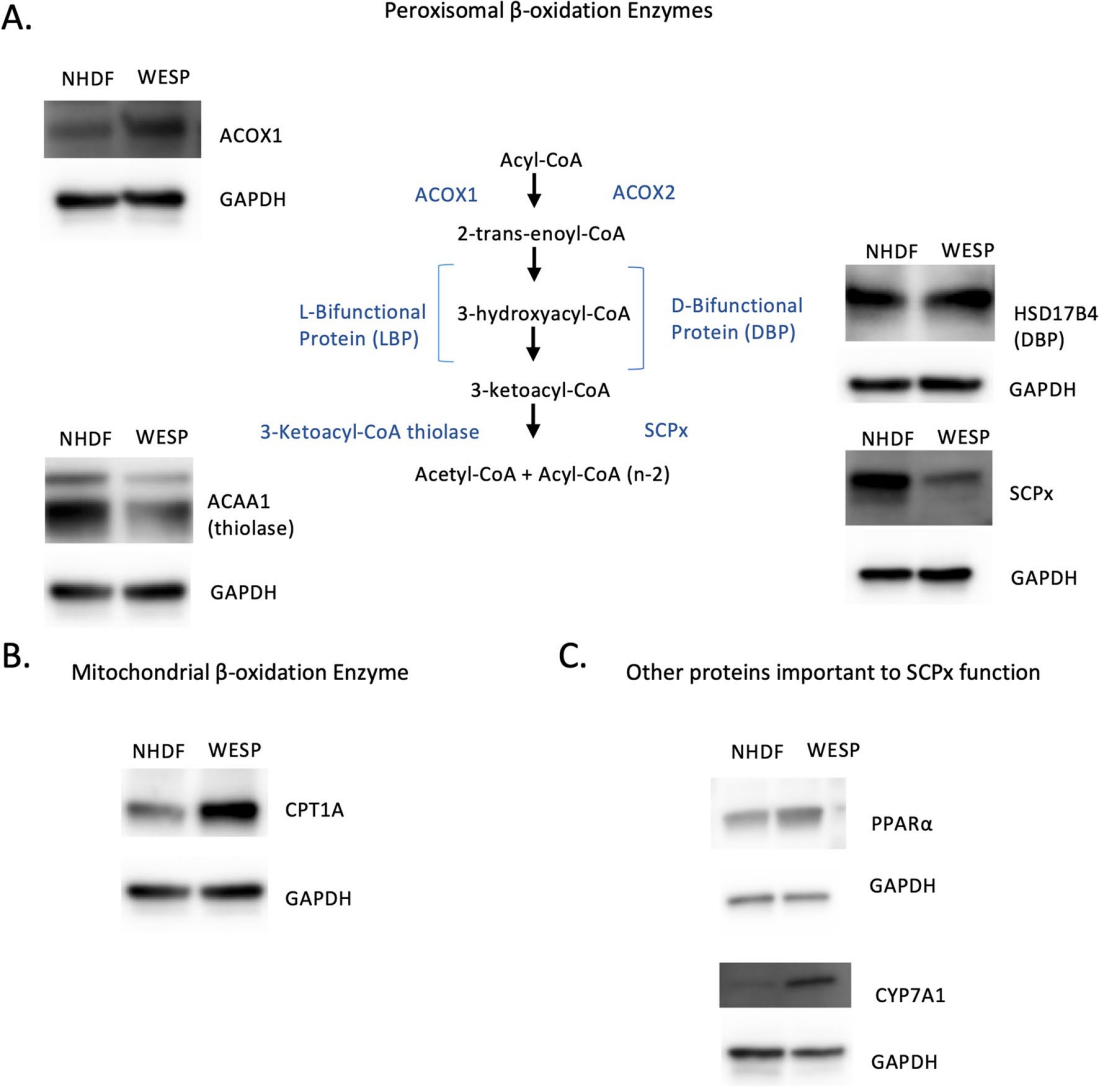
Western blot analyses of β-oxidation enzymes. **A** Western blot analyses of protein levels of ACOX1, ACAA1 (thiolase), HSD17B4 (DBP), and SCPx, mapped according to each protein’s role in the peroxisomal β-oxidation pathway. Left side of the pathway is the classical pathway and right shows the alternative pathway. **B** Western blot analyses of protein levels of CPT1A which is an important mitochondrial β-oxidation enzyme. **C** Western blot analyses of protein levels of PPARα and CYP7A1

We next wanted to identify any potential changes in other peroxisomal β-oxidation enzymes along the straight-chain fatty acyl-CoA pathway. Figure 3A demonstrates an increase in ACOX1 but a decrease in ACAA1 in patient WESP cells. In contrast, oxidation of 2-methyl-branched fatty acyl-CoAs was not affected as demonstrated by the lack of differences in DBP levels (Fig. 3A). These data suggest that the patient’s SCPx variant may be associated with alterations in the levels of other peroxisomal β-oxidation enzymes, which may affect the metabolism of fatty acids beyond those directly metabolized by SCPx.

As mitochondria are the other main site of fatty acid β-oxidation, we were also interested in measuring the levels of mitochondrial β-oxidation enzymes. Carnitine palmitoyltransferase 1 (CPT1A) is a key enzyme in the transport of long-chain fatty acids across the inner mitochondrial membrane. Immunoblot data showed that CPT1A levels were increased in patient fibroblasts compared to control (Fig. 3B). These data further suggest that the patient’s SCPx variant was accompanied by alterations in mitochondrial fatty acid β-oxidation in addition to its effects on peroxisomal β-oxidation.

To assess other putative pathways influenced by SCPx, we next examined levels of peroxisome proliferator-activated receptor α (PPARα), a nuclear receptor that regulates the expression of genes involved in fatty acid β-oxidation [18]. Immunoblotting data showed that PPARα was increased in patient fibroblasts compared to control cells, consistent with the increased expression of PPARα-responsive genes in *Scp2* gene-ablated mice (Fig. 3C) [19]. Additionally, given the recognized role of SCPx in bile acid synthesis, we next measured levels of cholesterol 7α-hydroxylase (CYP7A1), a rate-limiting step in this process [20]. We found that levels of CYP7A1 were increased in patient fibroblasts compared to control cells, which is in line with a previous report citing CYP7A1 repression in *SCP2*-overexpressing human hepatocytes (Fig. 3C) [21].

### RNA-sequencing identified differentially expressed genes and altered pathways related to lipid trafficking and metabolism

**Fig. 4:**
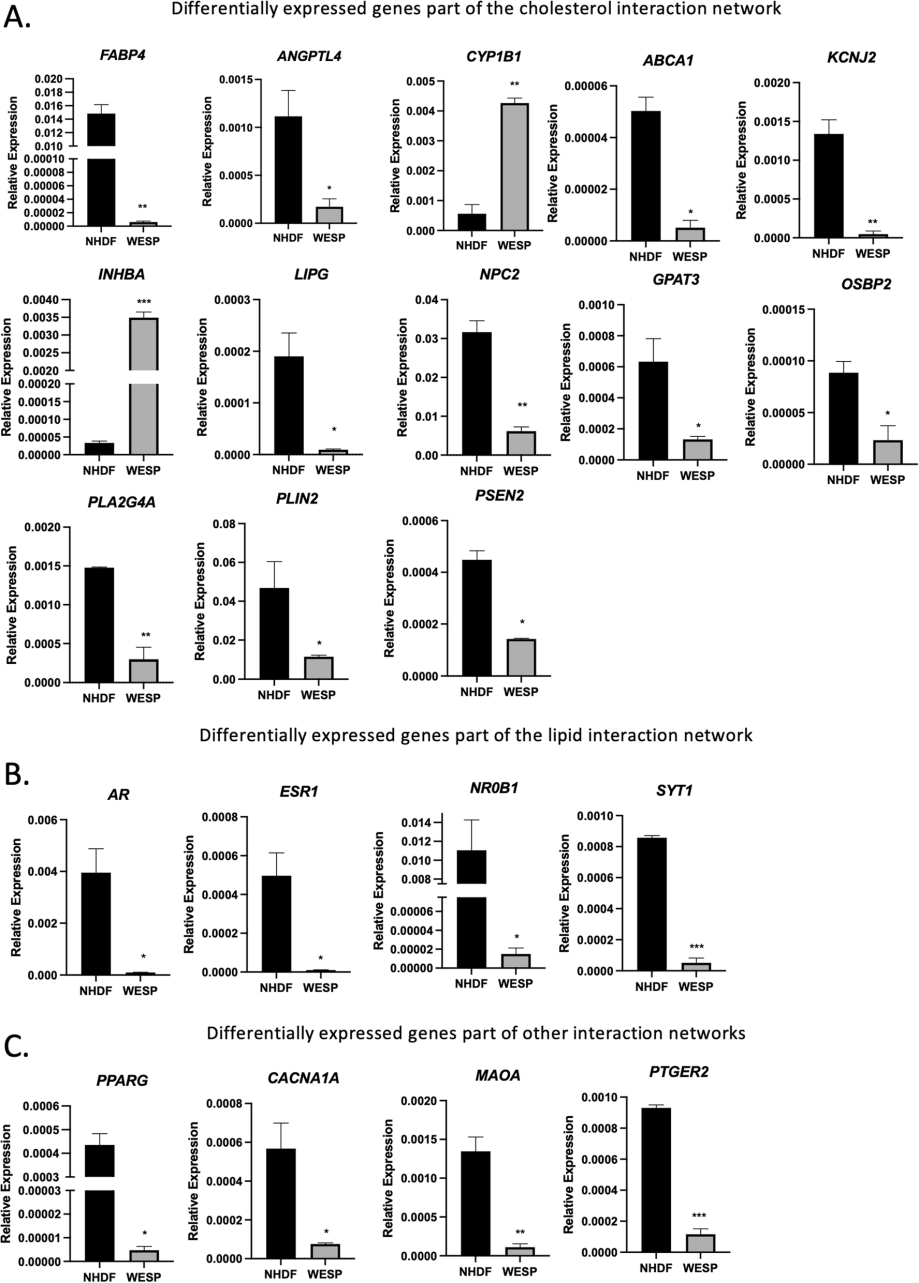
Confirmation of differentially expressed genes identified by RNA sequencing. **A** qRT-PCR analyses of differentially expressed genes part of the “cholesterol” interaction network. **B** qRT-PCR analyses of differentially expressed genes part of the “lipid” interaction network. **C** qRT-PCR analyses of differentially expressed genes part of other interaction networks. Data are shown as mean ± SEM (*n* = 3). *p < 0.05; **p < 0.01; ***p < 0.001

The observed effects on fatty acid β-oxidation and on bile acid synthesis prompted us to seek a broader view of alterations in the patient’s transcriptome. To this end, we sought to identify differential expression of genes governing lipid transport and metabolism. RNA sequencing data were confirmed by RT-qPCR of several lipid-related genes (Fig. 4). We uploaded our DEG list generated through Partek Genomic Flow into Ingenuity Pathway Analysis (IPA). Overlaying the DEG list with the “cholesterol” interaction network identified significantly decreased expression of: *FABP4* [MIM: 600434]*, ANGPTL4* [MIM: 605910]*, ABCA1* [MIM: 600046]*, KCNJ* [MIM: 601534], *LIPG* [MIM: 603684]*, NPC2* [MIM: 601015]*, GPAT3* [MIM: 610958]*, OSBP2* [MIM: 606729]*, PLA2G4A* [MIM: 600522]*, PLIN2* [MIM: 103195]*,* and *PSEN2* [MIM: 600759]*.* In contrast, *CYP1B1* [MIM: 601771] and *INHBA* [MIM: 147290] were significantly increased in WESP cells compared to NHDF (Fig. 4A). We pursued a similar strategy in addressing the “lipid” interaction network. This strategy yielded significantly reduced expression of *AR* [MIM: 313700]*, ESR1* [MIM: 133430]*, NR0B1* [MIM: 300473]*,* and *SYT1* [MIM: 185605] in WESP cells compared to NHDF (Fig. 4B). This process was also done with other interaction networks including that of phospholipids, *SCP2*, testosterone, and steroidogenic acute regulatory protein (STAR), which is known to play a critical role in cholesterol transport for steroidogenesis [22]. These yielded the identification of many genes that were also found in our cholesterol and lipid interaction searches, but also the addition of *PPARG* [MIM: 601487] for the phospholipid interaction network, *CACNA1A* [MIM: 601011] for the *SCP2* interaction network, *MAOA* [MIM: 309850] for the testosterone interaction network, and *PTGER2* [MIM: 176804] for the STAR interaction network, each of which were decreased in WESP cells compared to NHDF (Fig. 4C).

To identify pathways that may be altered due to changes in the patient’s transcriptome, we uploaded the DEG list that was confirmed by RT-qPCR into MetaboAnalyst 5.0. This online platform mapped the DEG list onto affected pathways (Table 3). The top affected pathway identified by MetaboAnalyst 5.0 was cholesterol metabolism, which aligns with known functions of SCPx in metabolism of cholesterol for bile acid synthesis and in cholesterol transport. The genes that are part of the cholesterol metabolism pathway that were confirmed to be differentially expressed between NHDF and WESP cells were *ABCA1, LIPG, ANGPTL4,* and *NPC2*. Another pathway that may be affected due to alterations in the expression of several genes is PPAR signaling, which is an important regulator of many genes critical for peroxisomal β-oxidation. DEG in this pathway included *FABP4, ANGPTL4, PLIN2*, and *PPARG*. Additionally, another pathway with many genes shown to be differentially expressed was that of serotonergic synapse signaling. DEG in this pathway included *CACNA1A, PLA2G4A*, and *MAOA*.

**Table 3:** Summary of RNA-sequencing-derived pathways

Pathway	Gene
Cholesterol metabolism	*ABCA1*
	*LIPG*
	*ANGPTL4*
	*NPC2*
PPAR signaling	*FABP4*
	*ANGPTL4*
	*PLIN2*
	*PPARG*
Serotonergic synapse	*CACNA1A*
	*PLA2G4A*
	*MAOA*

### Alterations in free fatty acid levels and related pathways

**Fig. 5:**
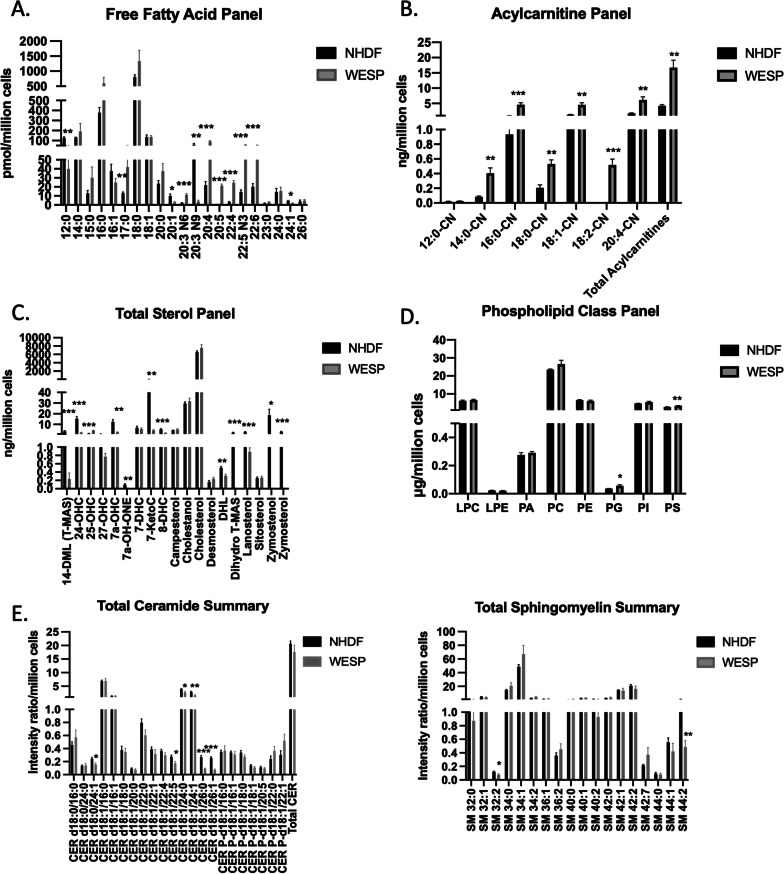
Lipidomic analyses of various lipid groups between NHDF and WESP cells. **A** Fatty acid profile. **B** Acylcarnitine profile. **C** Sterol profile. **D** Phospholipid profile. **E** Sphingolipid profile: Ceramides (left) and Sphingomyelins (right). Data are shown as mean ± SEM (*n* = 5). *p < 0.05; **p < 0.01; ***p < 0.001

The identification of many altered fatty acids in the patient’s plasma fatty acid profile and of many lipid-related pathways from our RNA sequencing analysis prompted us to investigate other potential changes in the patient’s lipidome. We conducted lipidomic analysis using NHDF and WESP cells for different classes of lipids including free fatty acids, acylcarnitines, sterols, phospholipids, and sphingolipids. Significant differences in the levels of many free fatty acid species identified through lipidomic analysis were consistent with the abnormalities in several fatty acid species derived from the patient’s plasma. For example, arachidonic acid (20:4), mead acid (20:3w9), and DHA (22:6) were out of normal range or altered in both the patient’s plasma and his fibroblasts (Table 1 and Fig. 5A). However, there were also several fatty acids that were significantly altered in WESP cells that were not detected in the patient’s plasma fatty acid profile. These included lauric acid (12:0), eicosatrienoic acid (20:3), EPA (20:5), DTA (22:4), and DPA (22:5) (Table 1 and Fig. 4A). Additionally, we noted that nervonic acid (24:1) was higher than normal in the patient’s plasma profile, but significantly lower than that seen in NHDF in our fibroblast lipidomic analysis. The discrepancies between the patient’s plasma fatty acid profile and the cellular lipidomic data may be attributed to differing extra- and intra-cellular levels of these lipids. Nevertheless, the significant differences in many species of free fatty acid levels between NHDF and WESP cells may further point to the pivotal role of SCPx in fatty acid metabolism.

To further identify potential pathways that could be affected as a result of perturbed free fatty acids, we used MetaboAnalyst 5.0 to map the lipidomic data onto pathways (Table 4). One of the pathways that involves several free fatty acids that were significantly altered between NHDF and WESP cells is the biosynthesis of unsaturated fatty acids. This pathway included EPA (20:5), DPA (22:5), DHA (22:6), eicosatrienoic acid (20:3 N9), arachidonic acid (20:4), adrenic acid (22:4), and icosenoic acid (20:1). Additionally, the linoleic acid metabolism pathway was altered with changes in the levels of arachidonic acid (20:4) and eicosatrienoic acid (20:3 N9). In addition to these lipids, *PLA2G4A*, which was part of our DEG list, is also part of this pathway. These data further suggest that the metabolism of several fatty acids is associated with reduced SCPx.

**Table 4:** Summary of lipidomics-derived pathways

Lipid class	Pathway	Lipid	Gene	Direction	*p* value/fold change
Sterol	Steroid biosynthesis	24,25-Dihydrolanosterol		Decreased	0.0056
		Lanosterol		Decreased	< 0.0001
		14-Demethyl-lanosterol		Decreased	0.0004
		Zymosterol		Decreased	0.0001
		Zymostenol		Decreased	0.0106
	Primary bile acid biosynthesis	25-Hydroxycholesterol		Increased	< 0.0001
		7alpha-Hydroxycholesterol		Decreased	0.0025
		7alpha-hydroxy-4-cholesten-3-one		Decreased	0.0013
Free fatty acid	Biosynthesis of unsaturated fatty acids	20:5 (Eicosapentaenoic acid)		Increased	< 0.0001
		22:5 (Docosapentaenoic acid)		Increased	< 0.0001
		22:6 (Docosahexaenoic acid)		Increased	0.0001
		20:3 N9 (Eicosatrienoic acid)		Decreased	0.0037
		20:4 (Arachidonic acid)		Increased	< 0.0001
		22:4 (Adrenic acid)		Increased	< 0.0001
		20:1 (Icosenoic acid)		Decreased	0.0199
	Linoleic acid metabolism	20:4 (Arachidonic acid)		Increased	< 0.0001
		20:3 N9 (Eicosatrienoic acid)		Decreased	0.0037
			*PLA2G4A*	Decreased	– 8.14
Phospholipid	Glycerophospholipid metabolism	Phosphatidylglycerol		Increased	0.0189
		Phosphatidylserine		Increased	0.0072
			*PLA2G4A*	Decreased	– 8.14
			*GPAT4*	Decreased	–10.64
Acylcarnitine	Fatty acid degradation	CAR 16:0 (L-palmitoylcarnitine)		Increased	0.0004

*p* values are shown for lipids and fold changes are shown for genes

### Alterations in acylcarnitine levels and related pathways

Since SCPx is known to play a critical role in fatty acid β oxidation and because we noted differences in levels of mitochondrial fatty acid β oxidation enzymes between NHDF and WESP cells, we next turned our attention to acylcarnitine. We found that the levels of several acylcarnitine species were significantly increased in WESP cells compared to NHDF (Fig. 5B). Consistent with this observation, MetaboAnalyst 5.0 identified CAR 16:0 as part of the fatty acid degradation pathway (Table 4). These data suggest that the *SCP2* variant may also be associated with alterations in mitochondrial fatty acid β oxidation, since acylcarnitines are required for fatty acid transport into mitochondria for β oxidation.

### Alterations in sterol levels and related pathways

As a known sterol transfer protein and important enzyme in cholesterol metabolism, we were also interested in identifying potential changes in sterol levels between NHDF and WESP cells. Lipidomic analysis identified that the levels of 14-demthyl-lanosterol, 24-hydroxycholesterol, 7α-hydroxycholesterol, 7-ketocholesterol, 8-dehydrocholesterol, 24,25-dehidrolanosterol, 7α-hydroxy-4-cholesten-3-one, 4,4-dimethyl-cholest-8(9)-en3β-ol, zymostenol, and zymosterol were all significantly lower, while 25-hydroxycholesterol was significantly higher in WESP cells compared to NHDF (Fig. 5C). These alterations in sterol levels are consistent with the known role of SCPx in sterol transport and metabolism.

We also identified pathways that may be altered due to the changes in sterol levels in patient fibroblasts (Table 4). Interestingly, one of the affected pathways was steroid biosynthesis, which included 24,25-dehidrolanosterol, lanosterol, 14-demthyl-lanosterol, zymostenol, and zymosterol. The alteration in the levels of these sterols that are part of the steroid biosynthetic pathway may be associated with the hypogonadism seen in this patient. Another pathway that was significantly altered by the changes in sterol levels is primary bile acid biosynthesis. This pathway involves 25-hydroxycholesterol, 7α-hydroxycholesterol, and 7α-hydroxy-4-cholesten-3-one. These data are consistent with the role of SCPx in cholesterol metabolism for bile acid synthesis.

### Alterations in phospholipid levels and related pathways

As indicated earlier, SCPx may play a role in the transport and/or metabolism of phospholipids [8, 12]. Our lipidomic analysis found that the amount of phosphatidylglycerol (PG) and phosphatidylserine (PS) were significantly higher in WESP cells compared to NHDF (Fig. 5D). These phospholipids are part of the glycerophospholipid metabolism pathway, in addition to *PLA2G4A* and *GPAT4* from our DEG list (Table 4). These data are aligned with the putative role of SCPx in phospholipid metabolism.

### Alterations in sphingolipid levels

We were also interested in identifying alterations in levels of sphingolipids since certain sphingolipids are highly enriched in the central nervous system and play critical roles in neuronal growth and differentiation. Additionally, abnormal levels of sphingolipids such as ceramides are often found in patients with neurodegeneration [23]. We found that many ceramide species had varying levels between control NHDF fibroblasts and patient WESP fibroblasts including CER d18:0/24:1, CER d18:1/22:5, CER d18:1/24:0, CER d18:1/24:1, CER d18:1/26:0, and CER d18/26:1 (Fig. 5E). Each of these ceramide species had significantly lower levels in patient fibroblasts compared to control. Because ceramide levels are determined in part by the ceramide/sphingomyelin cycle in which ceramide can be both produced from sphingomyelin or metabolized to sphingomyelin, we also measured sphingomyelin levels in both cell types [24]. Interestingly, while there were many species of ceramides that were decreased in WESP cells compared to NHDF, there were only two sphingomyelin species that had significantly different levels SM 32:2 and SM 44:2 (Fig. 5E). This suggests that, although the ceramide/sphingomyelin cycle may be functioning normally, there may be alterations in other pathways of ceramide production, such as from de novo synthesis or from the ceramide salvage pathway [24]. The varying levels in sphingolipids between control and patient cells also suggest that altered ceramide levels may play a role in the patient’s neurodegenerative phenotype. Collectively, our lipidomic data show that SCPx deficiency is accompanied by reorganization of the patient’s lipidome, which may provide insight into some of the patient’s clinical presentations.

### Identification of compounds that increase SCPx expression

**Fig. 6:**
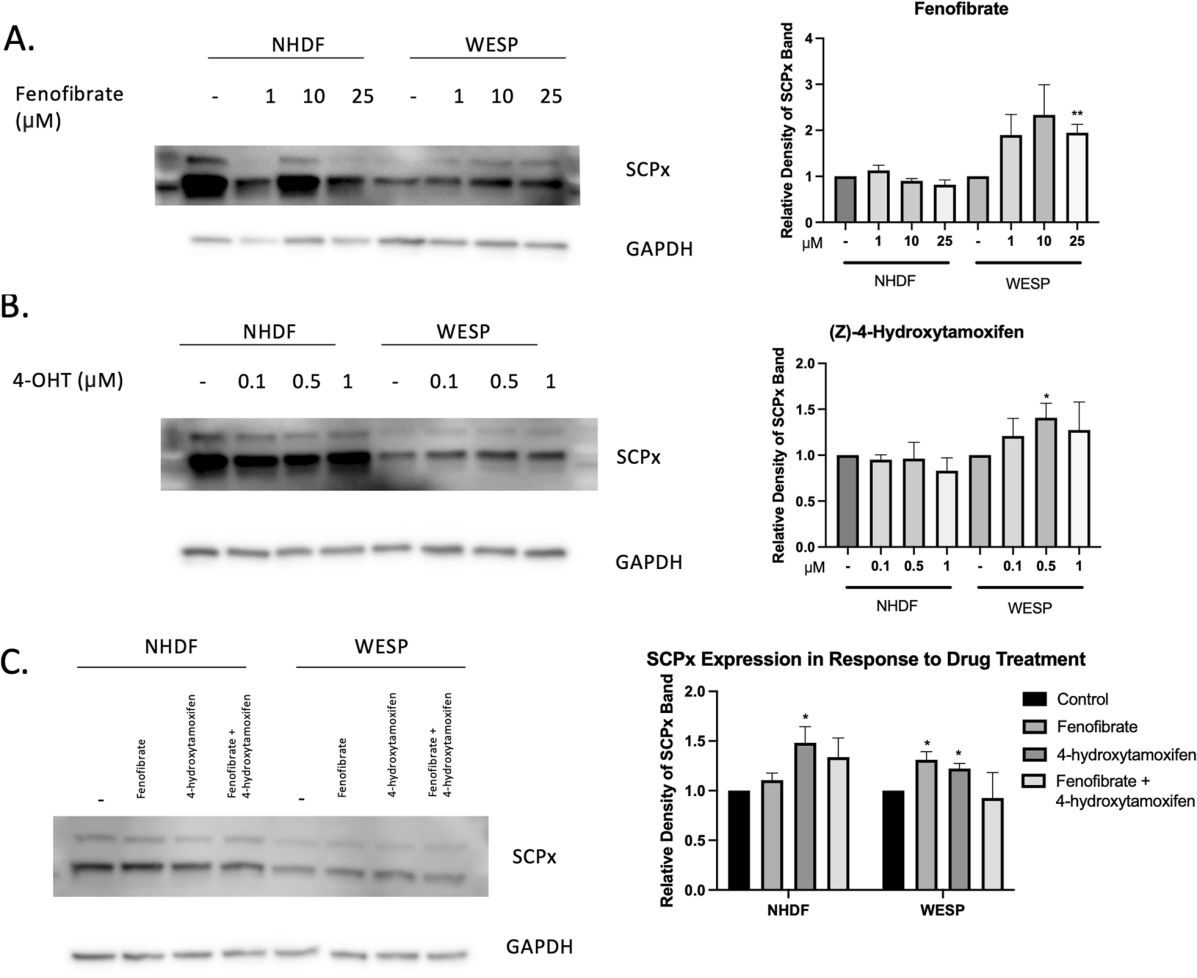
SCPx protein levels in WESP cells in response to fenofibrate and 4-hydroxytamoxifen treatment. **A** Western blot analyses of samples treated with various concentrations of fenofibrate for 24 h (left) and quantification of the blots (right). **B** Western blot analyses of samples treated with various concentrations of 4-hydroxytamoxifen (4-OHT) for 24 h (left) and quantification of the blots (right). **C** Western blot analyses of samples treated with 25 µM fenofibrate, 0.5 µM 4-OHT, and the fenofibrate + 4-OHT combination for 24 h (left) and the quantification of the blots (right). All Western blots were conducted in triplicate. Quantification was done using ImageJ analyses after normalizing to the untreated band for each group followed by normalizing to GAPDH. Data are shown as mean ± SEM (*n* = 3). *p < 0.05; **p < 0.01

Because the patient’s *SCP2* mutation results in decreased SCPx expression and is associated with alterations in lipid metabolism, we screened for compounds that could restore SCPx levels. Prompted by our differential expression profiling pointing to possible PPAR involvement, we treated patient and control fibroblasts with fenofibrate, a widely adopted PPARα agonist for treatment of dyslipidemia [25]. Unlike in normal fibroblasts which were relatively unaffected, patient WESP cells showed an increase in SCPx levels following fenofibrate treatment (Fig. 6A). As a previous report also showed that the selective estrogen receptor modulator 4-hydroxytamoxifen (4-OHT), also known as afimoxifene, can increase SCPx expression in DL23 cells [26], we also tested this compound. We found that 4-OHT treatment significantly and selectively increased SCPx levels compared to untreated patient cells (Fig. 6B). These data suggest that that 4-OHT may also be a viable strategy for increasing SCPx levels in conditions characterized by SCPx deficiency.

Since fenofibrate and 4-OHT have separate mechanisms of action, we hypothesized that a combination treatment of the two may have synergistic effects in increasing SCPx expression. However, treating patient fibroblasts with fenofibrate and 4-OHT resulted in no additional increases from those observed with either compound alone (Fig. 6C). This shows that, while these drugs can independently increase SCPx levels, they do not work synergistically on this target.

### Fenofibrate and 4-OHT improve the patient’s fatty acid profile

**Fig. 7:**
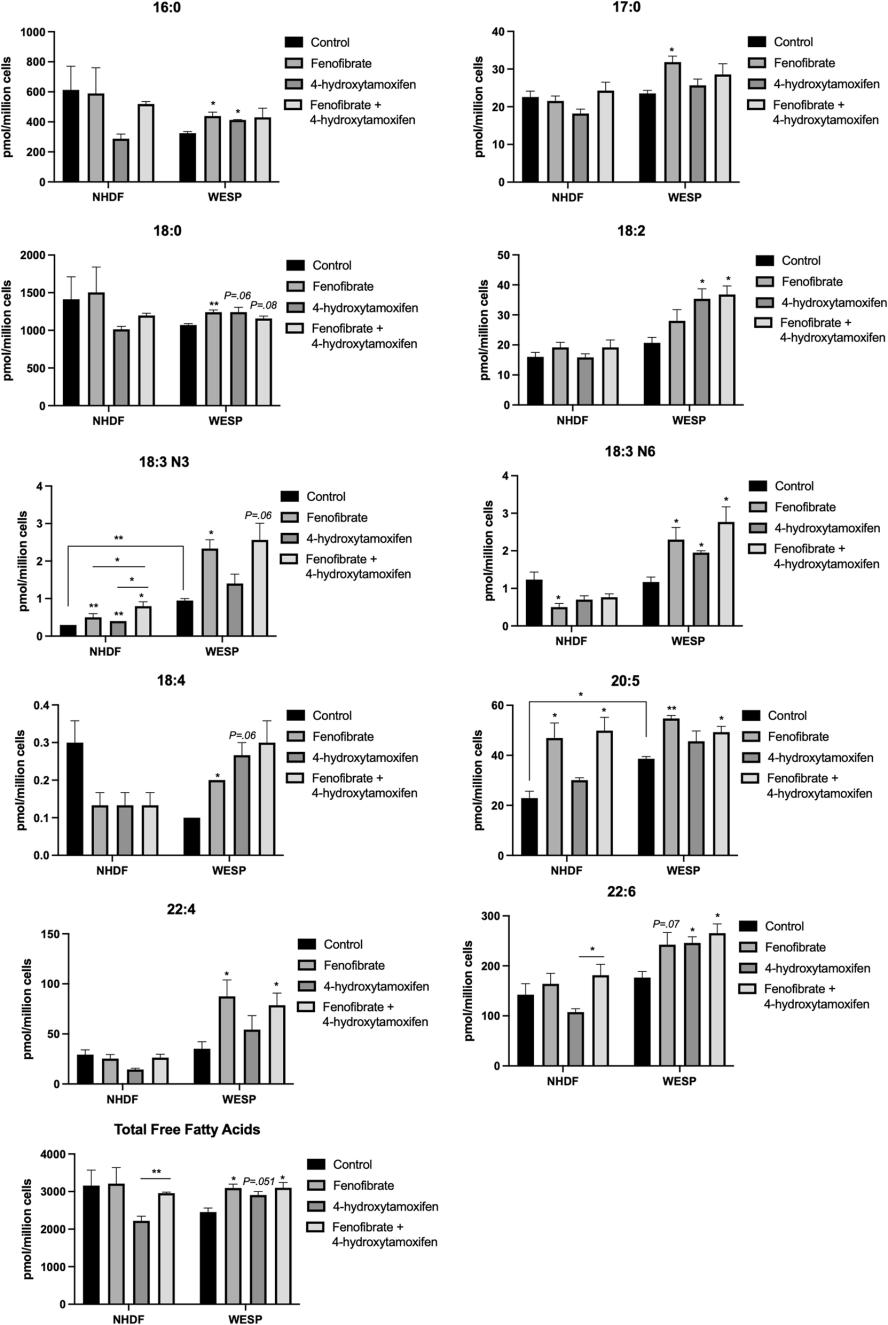
Fatty acid profile of NHDF and WESP cells following treatment. Measurement of various species of free fatty acids after treatment with 25 µM fenofibrate, 0.5 µM 4-hydroxytamoxifen, or a combination of the two compounds for 24 h. Data are shown as mean ± SEM (*n* = 3). *p < 0.05; **p < 0.01; ***p < 0.001

Finally, we wanted to investigate whether increasing SCPx expression through fenofibrate or 4-OHT is sufficient to restore the patient’s free fatty acid levels. We analyzed free fatty acid levels in NHDF and WESP cells treated with fenofibrate alone, 4-OHT alone, or fenofibrate and 4-OHT in combination for 24 h. The complete fatty acid profiles including the levels of all species identified following treatments are detailed in Additional file 3. We confirmed that many fatty acid species in the patient’s cells were increased following treatment with fenofibrate, including 16:0, 17:0, 18:0, 18:2, 18:3 N3, 18:3 N6, 18:4, 20:5, and 22:4 (Fig. 7). Further, total free fatty acids in patient cells were also significantly increased by fenofibrate treatment. Similarly, 4-OHT treatment demonstrated a positive effect on fatty acid levels including: 16:0, 18:2, 18:3 N6, and 22:6 (Fig. 7). However, in general, 4-OHT was not as effective in increasing fatty acid levels as fenofibrate. Additionally, the combination of drugs did not exhibit a synergistic effect on fatty acid levels. Collectively, these data show that treatment with fenofibrate or 4-OHT alone can improve the fatty acid profile in patient cells and provide evidence that these compounds may be beneficial in the treatment of SCPx deficiency.

## Discussion

Here, we report a patient presenting with progressive neurodegeneration including loss of cranial nerve function affecting the patient’s ability to swallow, chew, speak, breathe, and control head movement. Additionally, the patient has history of primary hypogonadism with azoospermia. Upon whole-genome sequencing, genetic variants were identified in the following genes: *LRRK1*, *MANBA*, *CLCN5*, *CACNA1S*, and *SCP2*. *LRRK1* encodes a leucine-rich repeat kinase shown to function in the regulation of bone resorption [27]. Mutations in *LRRK1* have been shown to cause osteosclerotic metaphyseal dysplasia [MIM: 615198], a rare skeletal dysplasia characterized by osteosclerosis of the long bones [27, 28]. In addition, one report using whole exome sequencing in two Parkinson’s disease (PD) patients identified variants in *LRRK1* (among other genes) [29]. However, a causative relationship was not shown since protein expression was unaffected. Next, mutations in *MANBA*, which encodes β-mannosidase, have been found to cause β-mannosidosis [MIM: 248510], a lysosomal storage disorder most commonly characterized by mental retardation, hearing loss, and frequent infections [30, 31]. The *CLCN5* gene encodes a voltage-gated chloride ion channel, and mutations in this gene have been associated with several diseases all characterized by progressive proximal renal tubulopathy including Dent disease [MIM: 300009], X-linked recessive nephrolithiasis [MIM: 310468], X-linked recessive hypophosphatemia rickets [MIM: 300554], and low molecular weight proteinuria [MIM: 308990] [32–34]. Mutations in the *CACNA1S* gene, which encodes a voltage-dependent calcium channel, cause hypokalemic periodic paralysis, type 1 [MIM: 170400], most classically characterized by episodic weakness with low serum potassium levels [35–37]. Lastly, mutations in *SCP2* have been reported to cause leukoencephalopathy with dystonia and motor neuropathy [MIM: 613724] and azoospermia [3]. Thus, based on this analysis of the variants, we have hypothesized that the most plausible explanation of the patient’s clinical phenotype would be due to the variant in *SCP2*, since the phenotype of the patient described here aligns closely to that of the previously described patients with mutations in *SCP2*. Although we cannot rule out the association of the other variants with the patient’s phenotype, which would also need to be formally tested, here we have focused solely on the potential contribution of *SCP2* to the patient’s clinical phenotype.

We report a third patient with SCPx deficiency and the first resulting from a heterozygous variant in *SCP2*. Both previously reported patients with SCPx deficiency were similar to each other, displaying cerebellar presentations and hypointensity in the pons and thalamus [3, 4]. Previously reported patients also had mutations on both alleles of the *SCP2* gene resulting in premature stop codons and a total absence of SCPx as visualized by Western blotting. Unlike these previous cases, the patient described herein lacks a cerebellar phenotype, and all imaging studies were unremarkable. Instead, our patient exhibited striking brainstem neurodegeneration with testicular spermatogenesis defects. Although the patient has a distinct neurological phenotype, our data showed that his heterozygous mutation in *SCP2* led to a decrease, but not the absence, of SCPx. Additionally, although previous cases of severe SCPx deficiency are associated with accumulation of the branched chain fatty acids pristanic and phytanic acid, our patient with reduced SCPx did not exhibit this feature [3, 4, 13, 38–41]. This suggests that the reduced SCPx that is produced, albeit at lower levels, maintains some activity, which may explain some differences between our patient and the previously reported cases.

Interestingly, we found that although the identified variant is a missense mutation, there was a significant decrease in SCPX mRNA levels. Previous reports have argued that single nucleotide polymorphisms (SNPs) and missense mutations can alter mRNA structure and are associated with changes in the levels and lengths of mRNA [42, 43]. It is possible that the variant substantially changed mRNA structure, leading to a pause in transcription and excess mRNA degradation. Our work also suggests that the patient’s heterozygous variant in *SCP2* was associated with extensive alterations in the patient’s transcriptome and lipidome. While it is not as common for heterozygous mutations in genes important for metabolic pathways to contribute to such widespread change, it is possible that the *SCP2* variant led to haploinsufficiency or a dominant negative effect. Since SCPx plays a critical role in the metabolism of a wide variety of lipids, it is plausible that a reduction in SCPx levels may lead to an accumulation of harmful products or a reduction of vital metabolites. Indeed, haploinsufficiency of glucose transporter 1 (*GLUT1*), important for glucose transport across the blood–brain barrier, leads to an insufficient amount of glucose in the brain and a cognitive development disorder [44].

We also found that, in addition to SCPx, other fatty acid β oxidation proteins had altered levels between control and patient fibroblasts. Consistent with this, previous work in SCPx-null mice has increases in β-oxidation enzymes in the absence of SCPx [1, 38]. Additionally, PPARα is known to regulate many genes in the fatty acid oxidation pathway [38, 45]. The increase in PPARα levels in WESP cells shown here may be responsible for the observed increases in ACOX1 and CPT1A. The latter being recognized targets of PPARα action [46–50]. However, we also noted a decrease in ACAA1, which is consistent with its negative regulation by PPARα [51]. Because ACAA1 is responsible for catalyzing the same step of the β-oxidation pathway as SCPx (but with different substrates), the decrease in ACAA1 levels may further explain the severity in the phenotype associated with SCPx deficiency, since both peroxisomal β-oxidation pathways are affected [39]. In addition to PPARα and the β-oxidation enzymes, we also found that levels of CYP7A1 are increased in WESP cells compared to NHDF, which is consistent with what was seen in *SCP2*-deficient mice [52]. Although fibroblasts do not synthesize bile acids, these data provide insight into what may be found when studying more relevant tissues.

Our RNA sequencing and lipidomic data identified several metabolic pathways that have genes and/or lipids that are altered between NHDF and WESP fibroblasts. Some of these pathways include steroid biosynthesis and serotonergic signaling, which are relevant to the patient’s testicular and neurological phenotypes, respectively. Cholesterol is the precursor of all steroid hormones, including testosterone, and thus, alterations in the metabolism of cholesterol may affect steroid biosynthesis and lead to low testosterone levels, accounting for the patient’s hypogonadism [53]. The patient’s low testosterone levels may also explain his azoospermia, since testosterone is also necessary for spermatogenesis and male fertility [54, 55]. Another pathway that was identified and may provide insight into the patient’s phenotype was serotonergic signaling, which included many downregulated genes. Alterations in serotonin signaling may contribute to various neurodevelopmental disorders [56]. Additionally, previous studies have suggested that inducing serotonergic signaling may be beneficial in slowing the progression of other neurodegenerative diseases such as Alzheimer’s disease [MIM: 104300] [57, 58]. Thus, altered serotonergic signaling may explain certain aspects of the patient’s neurodegenerative phenotype. Taken together, our gene expression, lipidomic, and pathway analyses provide further insight into the biochemical basis of the patient’s striking clinical presentation.

There are several reasons for which the *SCP2* variant may have resulted in a widespread alteration of genes and lipids contributing to the clinical presentations seen in the patient. Here, we showed that the *SCP2* variant resulted in a reduction of the levels of some lipids and an accumulation of other lipids, which may result in a decreased supply or toxic levels of those lipids, respectively. Firstly, defects in β-oxidation may reduce the supply of energy needed by certain tissues. For example, fatty acids are used for the production of ketone bodies, which are a vital source of fuel for the brain to spare glucose for other uses [59]. With reduced supply of ketone bodies, glucose utilization may increase, further decreasing the availability of glucose to support major processes [59]. Although poorly understood, it has been suggested that reprogramming of certain tissues to rely on glucose for energy when fatty acids are unavailable is mediated by widespread alterations in gene expression [60]. Additionally, decreased hepatic ATP production may lead to hyperammonemia which causes defects in the urea cycle, resulting in encephalopathy [59]. Further, decreased energy supply to skeletal muscle and cardiac muscle, which rely heavily on the β-oxidation of long-chain fatty acids for energy, results in muscle weakness or hypotonia and hypertrophy of the myocardium, respectively [59]. Another explanation for the patient’s presentations given the data shown here is toxicity due to an accumulation of metabolites. Many intermediates of β-oxidation have been reported to be toxic including C8-C10 and 3-hydroxy C12-C16 [59]. An accumulation of these metabolites has been shown to have several toxic effects such as increased ROS generation, uncoupled oxidative phosphorylation, and interference with DHA metabolism which may result in clinical consequences including encephalopathy, muscle weakness, cardiac arrhythmias, polyneuropathy, and demyelination [61–66].

To begin to translate our findings to the bedside, we sought to identify compounds that could restore SCPx levels. Previous reports have shown that *SCP2* is a PPARα responsive gene and that some PPARα ligands can increase *SCP2* expression [67–69]. Additionally, based on our findings that the patient’s *SCP2* mutation is associated with a decrease in peroxisome abundance and to alterations in PPAR signaling, we hypothesized that treatment of patient fibroblasts with a PPARα agonist may be a viable intervention. Indeed, treatment with fenofibrate increased SCPx levels in patient WESP fibroblasts. Moreover, we identified that 4-OHT, a selective estrogen receptor modulator, also increased SCPx expression. While unclear, our patient’s cell responsiveness may be afforded by their endogenously downregulated *ESR1* gene expression. We also found that while fenofibrate or 4-OHT treatment induced SCPx levels in patient fibroblasts, normal fibroblasts were relatively unaffected. This may be due to a negative feedback mechanism on PPARα activity under normal conditions to control fatty acid oxidation as in control fibroblasts, but increased PPARα activity in response to fenofibrate when fatty acid oxidation is disrupted as in patient fibroblasts. Lastly, we identified that treatment with fenofibrate or 4-OHT improves the fatty acid profile of patient fibroblasts. It should be noted, however, that the sample size used for the data in Fig. 7 was smaller than that used in Fig. 5, which may explain differences in the control NHDF and WESP free fatty acid levels between the two data sets. Nevertheless, our data indicate a reorganization of the lipid profile of patient fibroblasts compared to control and point to the potential use of fenofibrate or 4-OHT as treatments for SCPx deficiency.

## Conclusions

In conclusion, our work describes the first patient with a heterozygous *SCP2* mutation resulting in reduced protein expression. The patient presented with azoospermia, cardiac dysrhythmia, muscle wasting, and brainstem neurodegeneration. This work suggests that a reduction in SCPx levels may contribute to widespread gene alterations and dysregulated lipid synthesis and metabolism. Our identification that fenofibrate and 4-OHT can restore SCPx protein levels and related functions should prompt future studies into their role for neurodegenerative peroxisomal disorders.

## Methods

### Genome sequencing

Whole-genome sequencing was performed on whole blood using next-generation sequencing (NGS) on the Illumina NovaSeq 6000 platform. 98% percent of the exome was sequenced at greater or equal to 10X depth coverage. Library preparation was performed using the Illumina TruSeq PCR-free library preparation kit according to the manufacturer’s instructions. Seven hundred micrograms of DNA was fragmented to 400 bp by acoustic shearing using a Covaris LE220 instrument (Covaris Inc., Woburn, MA). Library size range was assessed using a Bioanalyzer (Agilent Technologies, Santa Clara, USA) and quantified with a Kapa library quantification kit (Roche Sequencing and Life Sciences, Wilmington, MA, USA). Genomic libraries were loaded on an Illumina patterned flowcell and followed by cluster generation and 150-bases paired-end sequencing on Illumina HiSeq X platform to generate 90–100 g bases of raw data per library. Sequencing of the DNA was performed on a research basis at The Center for Applied Genomics (TCAG; Hospital for Sick Children, Toronto, ON, Canada).

Data processing and analysis was performed using 1) the Franklin Genoox Platform (genoox.com) and 2) local GATK best practices (GATK 3.7). Reads were aligned using Burrows-Wheeler Aligner (BWA) against the hg19 reference genome. Duplicate reads were removed. Variant calling was performed using GATK (version 4.1) and FreeBayes (version 1.1.0). 2). Base calling was performed using bcl2fastq 2.20 (for HiSeq 2500) or HiSeq Analysis software (for HiSeq X). Reads were mapped to the b37 reference genome using the BWA-mem algorithm, and duplicate reads were marked using Picard Tools. Local realignment and base quality score recalibration using GATK followed. Variants were called using GATK HaplotypeCaller. For whole-genome sequencing (WGS), variant quality score recalibration (VQSR) was performed for filtering variants. Variants were identified for further analysis. Human phenotype ontology (HPO) was used to query for genes related to the phenotypic presentation including terms for neurological, neuromuscular, and musculoskeletal search terms.

### Clinical samples

This individual was enrolled in the Adults with Undiagnosed Rare Disease genome sequencing research study approved by the Mount Sinai Hospital Research Ethics Board (#12–0222-E) [70]. These studies abide by the Declaration of Helsinki principles.

### Cell culture

Patient fibroblasts (WESP) were maintained in minimum essential medium α (MEM α) (Thermo Fisher Scientific, Waltham, MA, USA) supplemented with 10% fetal bovine serum (Sigma, St. Louis, MO, USA). Adult normal human dermal fibroblasts (NHDF) were acquired from PromoCell and grown in Fibroblast Growth Medium 2 with SupplementMix (PromoCell GmbH, Heidelberg, Germany). Fibroblasts were grown at 37 °C and 5% CO_2_.

Cells were treated with 1, 10, or 25 µM fenofibrate (Sigma, St. Louis, MO, USA) or 0.1, 0.5, or 1 µM (Z)-4-hydroxytamoxifen (Sigma-Aldrich, St. Louis, MO, USA) for 24 h prior to cell pellet collection for downstream analyses. For combination treatments, 25 µM fenofibrate and 0.5 µM (Z)-4-hydroxytamoxifen were used to treat the cells for 24 h prior to cell pellet collection.

### Next-generation sequencing

Amplicon-EZ sequencing was done in triplicate to confirm the c.572A > G mutation in WESP cells and to confirm that NHDF cells had wild-type (WT) *SCP2* (GENEWIZ from Azenta Life Sciences, South Plainfield, NJ, USA). Basic Local Alignment Search Tool (BLAST) was used to align the WT human *SCP2* gene with next-generation sequencing results from WESP samples (National Center for Biotechnology Information, Bethesda, MD, USA).

### Quantitative real-time polymerase chain reaction

The Quick-RNA MiniPrep Plus kit (Zymo Research, Irvine, CA, USA) was used to extract total RNA for all qRT-PCR data shown. In total, 500 ng of extracted RNA per sample was used for reverse transcription using PrimeScript RT Master Mix (Takara Bio, Mountain View, CA, USA). qRT-PCR was done using Applied Biosystems PowerUP SYBR Green Master Mix (Thermo Fisher Scientific, Waltham, MA, USA) and tqTOWER^3^ (Analytik Jena AG, Jena Germany). Primer information is detailed in Additional file 2. Relative expression levels of genes were calculated by subtracting the cycle threshold value (Ct) from the detection limit (40 Ct) resulting in the ΔCt value, then by taking the log2 of the -ΔCt. Gene expression data were normalized to *GAPDH*.

### Immunoblot analysis

Immunoblot analysis was carried out as previously described [71]. Briefly, total protein was extracted from fibroblasts using RIPA buffer with Pierce Protease Inhibitor and protein concentration was measured using the Pierce BCA Protein Assay kit (Thermo Fisher Scientific). 7.5 µg of protein per sample, and a 4–20% Tris–glycine gradient gel were used for SDS-PAGE (Bio-Rad, Hercules, CA, USA). Protein was transferred to a polyvinylidene fluoride membrane. 5% bovine serum albumin in PBST was used for all blocking steps (Equitech-Bio, Kerrville, TX, USA). All primary antibodies were incubated in blocking solution overnight at 4 °C. Information about antibodies is detailed in Additional file 1. Secondary antibodies were incubated for 1 h at room temperature. Restore Western Blot Stripping Buffer was used to strip the membranes (Thermo Fisher Scientific). Membranes were visualized by Radiance Peroxide and Radiance Plus (Azure Biosystems, Dublin, CA, USA). Immunoreactive proteins were imaged using the Azure c600 (Azure Biosystems).

### Immunofluorescence

Fibroblasts were plated at a density of 200,000 cells per well on coverslips for 24 h. Cells were stained with 0.1 µM Mitotracker Red CMXRos for 30 min at 37 °C, washed with PBS 3 times, and fixed with 4% PFA for 10 min at room temperature (Thermo Fisher Scientific). Cells were washed, incubated in 0.1% Triton X-100 for 10 min at room temperature, and washed again. Samples were blocked with 5% donkey serum, washed, and incubated with primary antibodies overnight at 4 °C. After washing, secondary antibodies were added for 30 min at room temperature and then washed again. Cells were stained with DAPI and observed using a Zeiss LSM 880 confocal microscope. Quantification was done using ImageJ.

### RNA sequencing

Three replicates per sample were sent for total cell RNA sequencing. RNA extraction and preparation was done according to protocol using the Qiagen Allprep RNA/DNA isolation kit (Qiagen, Valencia, CA, USA). For library preparation, the Clontech Takara SMARTer Total RNA Stranded Pico V2 Library prep kit was used (Takara Bio). Libraries were dual index 8 bp and Illumina Adapters were used. The libraries were sequenced on a Nextseq500 at 2 × 75 cycles. Partek Genomics Suite was used to analyze the gene expression data and to identify differentially expressed genes between WESP and NHDF samples. Significantly differentially expressed genes were identified using FDR < 0.05 and fold change < –2 or > 2 as cutoffs.

### Lipidomic analyses

Five replicates of WESP cells and five replicates of NHDF cells with 4 million cells per sample were used for lipidomic analysis, which was performed at the UCSD Lipidomics Core. Lipidomic analytical procedures were conducted as described previously [72]. Briefly, for free fatty acid analysis, 500 µl MeOH was added to 50 µl of sample for extraction. This was followed by the subsequent addition of 25 µl 1 N HCl and 100 µl of 0.1 ng Internal Standard Mix of 12:0-d23,14:0-d27,15:0-d29,16:0-d31,18:0-d35,18:1-d17,18:2-d11,20:4-d11,20:5-d5, 22:6-d5, and 24:0-d47 (Cayman Chemical Company, Ann Arbor, MI, USA). The samples were vortexed and iso-octane was added. The samples were vortexed again, centrifuged, and the iso-octane layer was collected. The sample was re-extracted with iso-octane, combined with the initial extract, and solvent was removed. Samples were derivatized with PFBB and DIPEA. Solvent was removed and the samples were reconstituted in 50 µl iso-octane and transferred to an MS vial with insert. GC–MS analysis was done using the Agilent 6890 N gas chromatograph equipped with an Agilent 7683 autosampler (Agilent Technologies). Fatty acids were separated using a 15 m ZB-1 column (Phenomenex, Torrance, CA, USA) and monitored using SIM identification. Analysis was performed using MassHunter software [73].

Acylcarnitines were extracted by adding 250 µl butanol/methanol (3:1) and 100 µl 1X CN-18:0-d3 internal standard (1 ng/µl) to 50 µl of sample (Cayman Chemical Company). The samples were vortexed, and 250 µl heptane/ethyl acetate (3:1) and 250 µl 1% acetic acid in H_2_O were added. The samples were vortexed and centrifuged, and the upper layer was collected. Solvent was removed. The samples were reconstituted in 50 µl NP Buffer A (59/40/1 IPA/HEX, H_2_O containing 10 mM NH_4_oAC). For LC–MS analysis, the samples were reconstituted in 50 µl 90% MeOH and 0.1% formic acid. 10 µl per sample was injected into a Waters Acquity UPLC System interfaced with an AB Sciex 6500 QTrap mass spectrometer (Sciex, Framingham, MA, USA). A Phenomenex Kinetics C18 2.1 × 150 mm 1.7um column was used for chromatographic separation using a step gradient from 100% buffer A (100% 10 mM ammonium acetate in water to 100% buffer B (70/30 isopropanol/acetonitrile/10 mM ammonium acetate) for 10 min. Acylcarnitines were separated by mass spectrometry using MRM scans in positive mode consisting of precursors for Q1 and m/z 85 for Q3. Data analysis was done using Analyst and Multiquant software [74].

Sterols were extracted by adding 500 µl butanol/methanol (3:1) and 250 µl 1 N NaOH to 100 µl of sample. An internal standard mix of 25-Hydroxycholesterol-d6, Desmosterol-d6, and Campesterol-d6 was added to 50uL of homogenate (Avanti Polar Lipids, Birmingham, AL, USA). Samples were saponified for 1.5 h at 37C with a final concentration of 0.2 N KOH. Samples were extracted by modified BUME. Extracts were brought to dryness and taken up in 90% methanol in water and run on a Waters Acquity UPLC interfaced with an AB Sciex 6500 QTrap mass spectrometer equipped with an APCI probe. Source settings were: Curtain Gas = 20, Collision Gas = Medium, Ion Spray Voltage = 5500, Temperature = 400, GS1 = 25, NC = 1. A Phenomenex Kinetex C18 1.7uM 2.1 mm x 150 mm column was used for chromatographic separation. A 30-min step gradient was employed using 70/30 acetonitrile/water with 5 mM ammonium acetate as Buffer A and 50/50 acetonitrile/water with 5 mM ammonium acetate as Buffer B with a flow of 0.5 mL/min. The gradient started at 0%B for 2 min, ramped to 10%B over 4 min, 15%B over 9 min, 50%B over 11 min, 100%B over 2 min, then held at 100%B for 2 min. Sterol species were identified by mass spectrometry using 30 MRMs (multiple reaction monitoring) in positive mode (see supplement). Standard curves were obtained in parallel using identical conditions. Data analysis was performed with Analyst and Multiquant software packages [72, 74].

Samples for phospholipids were homogenized into 400uL of 10% methanol in water. A mix of PC 12:0/13:0, PE 12:0/13:0, PS 12:0/13:0, PI 12:0/13:0, PG 12:0/13:0, and PA 12:0/12:0 internal standard was added to 50uL of cell homogenate (Avanti Polar Lipids, Birmingham, AL, USA). Samples were extracted using a modified BUME method. The extracts were brought to dryness and reconstituted in Buffer A (59/40/1 isopropanol/hexane/10 mM ammonium acetate). The phospholipids were analyzed on a Waters Acquity UPLC interfaced with an AB Sciex 6500 QTrap mass spectrometer. A Phenomenex Silica 3uM 2.1 mm x 150 mm column was used for chromatographic separation using a step gradient from 100% buffer A to 100% buffer B (50/40/10 isopropanol/hexane/10 mM ammonium acetate) over 16 min. Phospholipid classes were separated by mass spectrometry using precursor ion (PI) and neutral loss (NL) scans in positive and negative ionization mode. Lipid classes were analyzed in Multiquant by selecting the TICs of the following scans for each class: PC, PIS m/z 184 Da ( +); PE, NLS 141 Da ( +); PS, NLS 185 Da ( +); PA, NLS 98 Da ( +); PG, NLS 172 Da ( +); and PI, PIS m/z 241 Da ( −). Lipid classes were quantified using whole class extracts run in parallel (Avanti Polar Lipids) [74, 75]. Ceramide and sphingomyelin analysis was derived from phospholipid extraction and analysis.

### Statistical analysis

GraphPad Prism 9 (GraphPad Software, San Diego, CA, USA) was used for statistical analyses and graphical presentation. Statistical analyses of data were performed using the Student's t test, and means were considered statistically different when *p* < 0.05. Lipids found to be statistically significantly different were uploaded to the metabolomics data analysis tool, MetaboAnalyst 5.0, for pathway analysis. All qPCR and immunoblot experiments were conducted in triplicate. Immunofluorescence data were analyzed with n = 10, RNA sequencing was analyzed using n = 3, initial lipidomic data were analyzed using n = 5, and lipidomic data following compound treatment were analyzed using n = 3. All data are presented as mean ± standard error of the mean.

## Supplementary Information

**Additional file 1**: Table of Information for Antibodies. Description of antibodies including protein target, name of antibody, manufacturer and catalog number, and dilution used. **Additional file 2**: Table of Primers. Description of primers used, including primer name and sequence. **Additional file 3**: Complete fatty acid profile of NHDF and WESP cells following treatment. Measurement of various species of free fatty acids after treatment with 25 µM fenofibrate, 0.5 µM 4-hydroxytamoxifen, or a combination of the two compounds for 24 hours. Data are shown as mean ± SEM (n=3). *p < 0.05; **p < 0.01; ***p < 0.001.

## Data Availability

The datasets supporting the conclusions of this article are included within this article and will be made available upon request or through the NCBI Gene Expression Omnibus (GEO) database: accession no.: GSE199634 (token for reviewers: ijibuwmidzabdqn) and NCBI’s ClinVar: accession no.: VCV001050589.2.

## Abbreviations

SCPx: Sterol carrier protein-x
*SCP2*: Sterol carrier protein 2
VLCFA: Very long-chain fatty acids
ACOX1: Acyl-CoA oxidase 1
LBP: L-bifunctional protein
ACAA1: 3-Ketoacyl-CoA thiolase
ACOX2: Acyl-CoA oxidase 2
DBP: D-bifunctional protein
AMACR: 2-Methylacyl-CoA racemace
PED: Peroxisomal enzyme deficiency
X-ALD: X-linked adrenoleukodystrophy
AMN: Adrenomyeloneuropathy
NHDF: Normal human dermal fibroblasts
NGS: Next-generation sequencing
BWA: Burrows-Wheeler Aligner
WGS: Whole-genome sequencing
VQSR: Variant quality score recalibration
HPO: Human phenotype ontology
BLAST: Basic Local Alignment Search Tool
PMP70: Peroxisomal membrane protein 70
CPT1A: Carnitine palmitoyltransferase 1
CYP7A1: Cholesterol 7α-hydroxylase
STAR: Steroidogenic acute regulatory protein
PG: Phosphatidylglycerol
PS: Phosphatidylserine
4-OHT: 4-Hydroxytamoxifen
PD: Parkinson’s disease
GLUT1: Glucose transporter 1
