# Supplementary material for: SCP2 variant is associated with alterations in lipid metabolism, brainstem neurodegeneration, and testicular defects
Source: Hum Genomics. 2022 Aug 22;16:32. doi: 10.1186/s40246-022-00408-w (PMC9396802; doi:10.1186/s40246-022-00408-w)
Supplement: Supplementary file 1 — Additional file 1: Table of Information for Antibodies. Description of antibodies including protein target, name of antibody, manufacturer and catalog number, and dilution used. Additional file 2: Table of Primers. Description of primers used, including primer name and sequence. Additional file 3: Complete fatty acid profile of NHDF and WESP cells following treatment. Measurement of various species of free fatty acids after treatment with 25 µM fenofibrate, 0.5 µM 4-hydroxytamoxifen, or a combination of the two compounds for 24 hours. Data are shown as mean ± SEM (n=3). *p < 0.05; **p < 0.01; ***p < 0.001. [file 40246_2022_408_MOESM1_ESM.pdf]

<u>Additional Files</u>

SCP2 variant is associated with alterations in lipid metabolism, brainstem neurodegeneration and testicular defects

Melanie Galano[1], Shereen Ezzat[2] and Vassilios Papadopoulos[1*]

[1] Department of Pharmacology and Pharmaceutical Sciences, School of Pharmacy, University of Southern California, Los Angeles, California, 90089, USA

[2] Department of Medicine, University of Toronto and Princess Margaret Cancer Center, Toronto, Ontario, M5G 2C1, Canada

Additional file 1. Table of Information for Antibodies

Protein Target	Name of Antibody	Manufacturer: Catalog No.	Species Raised in	Dilution Used (IB, IF)
SCPx	SCP2/SCPx Polyclonal Antibody	Proteintech: 14397-1-AP	Rabbit	1:1000 (IB) 1:400 (IF)
SCP2	SCP2 Polyclonal Antibody	Proteintech: 23006-1-AP	Rabbit	1:1000
ACOX1	ACOX1 Polyclonal Antibody	Proteintech: 10957-1-AP	Rabbit	1:1000
ACAA1	ACAA1 Polyclonal Antibody	Proteintech: 12319-2-AP	Rabbit	1:1000
HSD17B4 (DBP)	HSD17B4 Polyclonal Antibody	Proteintech: 15116-1-AP	Rabbit	1:1000
CPT1A	Anti-CPT1A Antibody	Abcam: ab128568	Mouse	1:1000
LCAD	ACADL-Specific Polyclonal Antibody	Proteintech: 17526-1-AP	Rabbit	1:1000
PPARα	Anti-PPAR alpha Antibody	Abcam: ab24509	Rabbit	1:1000
CYP7A1	Anti-Cyp7a1 Antibody	Sigma-Aldrich: MABD42	Mouse	1:1000
GAPDH	GAPDH (D4C6R) Mouse mAb	Cell Signaling Technology: 97166	Mouse	1:2000
PMP70	Anti-PMP70 antibody	Sigma-Aldrich: SAB4200181	Mouse	1:400 (IF)
Rabbit IgG	WesternSure^{TM} HRP Goat Anti-Rabbit IgG (H+L)	LI-COR: 926-80011	Goat	1:5000
Mouse IgG	WesternSure^{TM} HRP Goat Anti-Mouse IgG (H+L)	LI-COR: 926-80010	Goat	1:5000
Rabbit IgG	Donkey anti-Rabbit IgG (H+L), Alexa Fluor 488	Thermo Fisher: A-21206	Donkey	1:400 (IF)
Mouse IgG	Goat anti-Mouse IgG (H+L), Alexa Fluor 647	Thermo Fisher: A-21235	Goat	1:400 (IF)

IB: Immunoblot; IF: Immunofluorescence

Additional file 2. Table of Primers

Primer	Sequence
SCP2-F (SCPx)	TTGGCAGAAGAAGCAGGCAA
SCP2-R (SCPx)	CTGGCGGGCCATAAACAAAG
SCP2-F	CGGAAGCCGCCAGTTCTTTT
SCP2-R	CACCCAGGTGGCCTCTTTAC
FABP4-F	ACAGCACCCTCCTGAAAACT
FABP4-R	GTGGCAAAGCCCACTCCTAC
ANGPTL4-F	GAGTTGCTGCAGTTCTCCGT
ANGPTL4-R	AAACCACCAGCCTCCAGAGA
CYP1B1-F	GTCCCCAAGGGCAGCG
CYP1B1-R	TGGTGCCCATGCTGCG
CYP27A1-F	TTACAAGGCCAAGTACGGTCC
CYP27A1-R	AGTGGTGTCCTTCCGTGGT
ABCA1-F	AATTGCGAGCGAGAGTGAGT
ABCA1-R	CCGTGGCTGGTCATTAACTGT
INHBA-F	AGAGTGGGGACCAGAAAGAGAA
INHBA-R	GGTGGGGGAACTCCTCACTA
LIPG-F	CCCACCAGCTTTACACGGAT
LIPG-R	GGCAGGATCCAAACCTGTGA
NPC2-F	CAGGTTTGTCTTGTGACCGC
NPC2-R	CTCCATCCACAGAACCGCAG
GPAT3-F	ACTGCCTCTGAGGGTTACCT
GPAT3-R	ACAAATGCCTCCCTTCTGGG
OSBP2-F	TTAAAGCCCCTGCCTCTTCTG
OSBP2-R	GCCATTTCACCCTGATTTCTGTAG
PLA2G4A-F	ACGAACCCAAAGGCACTGAA
PLA2G4A-R	AGGAGTCCTGTGTGGCAAAG
PLIN2-F	GCTGCAGTCCGTCGATTTCT
PLIN2-R	TCTTCACACCGTTCTCTGCC
PSEN2-F	GCGGCAGAGCAGGCATTT
PSEN2-R	CACACAGCTCCCTCACATCA
KCNJ2-F	GCGCCAGCAACAGGACAT
KCNJ2-R	GGACTCCAGTGCTTCTGCTT
AR-F	GGGGACATGCGTTTGGAGA
AR-R	TTCCCTTCAGCGGCTCTTTT
ESR1-F	TGGGAATGATGAAAGGTGGGAT
ESR1-R	AGCATCCAACAAGGCACTGA
NR0B1-F	GTGCTCTTTAACCCGGACGTG
NR0B1-R	TCATCCATGCTGACTGTGCC
SYT1-F	TCCTGACCTGCTGCTTTTGT
SYT1-R	CATCCTTGAGGGCCTGATCT
PPARG-F	CTGGGGCGCTTGGGTC
PPARG-R	GTGTCAACCATGGTCATTTCGTT
CACNA1A-F	TGTCTGAACGGCTGGATGAC
CACNA1A-R	GGTCAAACTCCGTCCCAACT
MAOA-F	GGTGGCATTTCAGGACTATCT
MAOA-R	TGGGTTGGTCCCACATAAGC
PTGER2-F	GCTCCTTGCCTTTCACGATTT
PTGER2-R	ACAACAGAGGACTGAACGCA

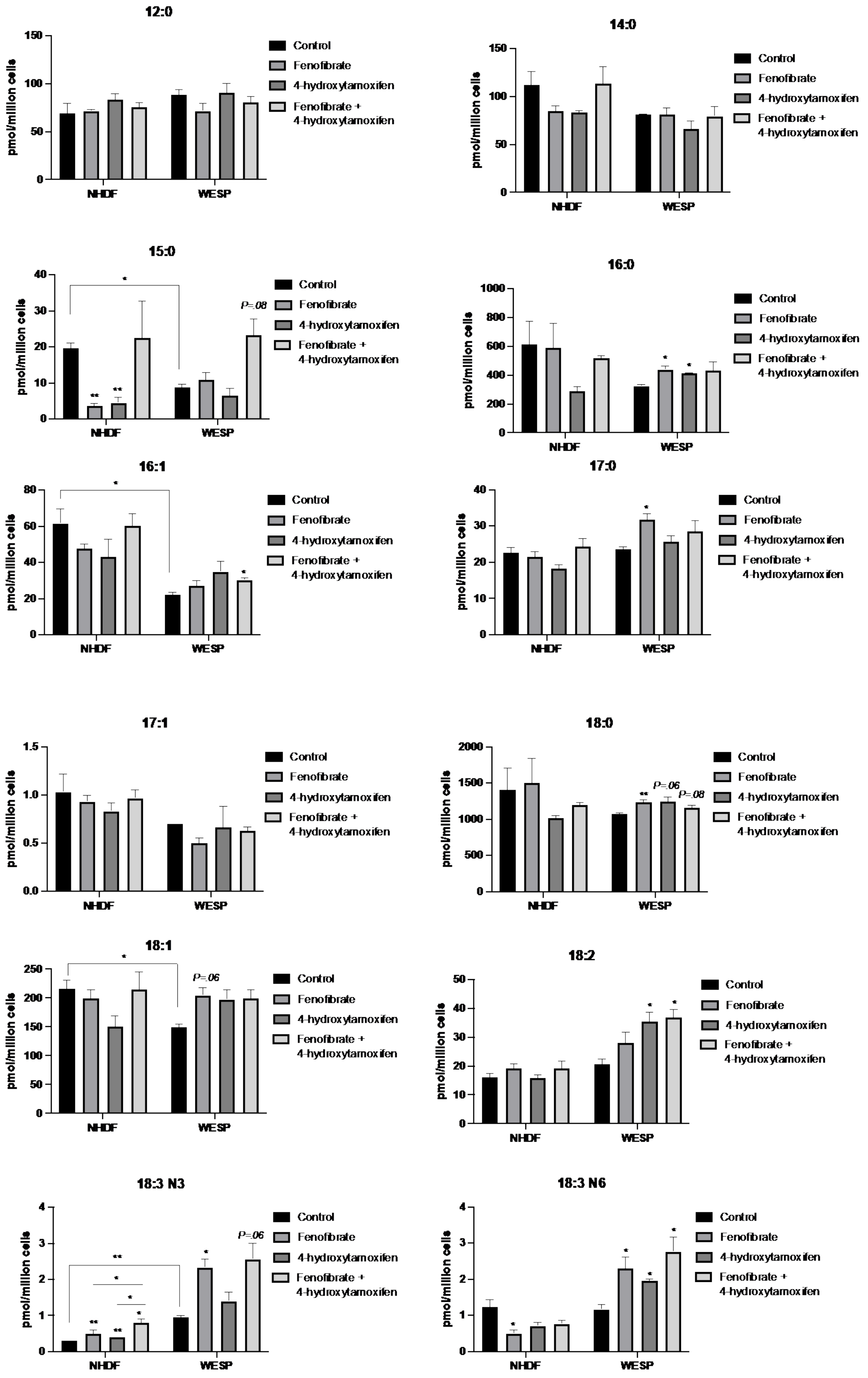
12:0
14:0
15:0
16:0
16:1
17:0
17:1
18:0
18:1
18:2
18:3 N3
18:3 N6
pmol/million cells
NHDF
WESP
Control
Fenofibrate
4-hydroxytamoxifen
Fenofibrate +
4-hydroxytamoxifen
P=.08
P=.06

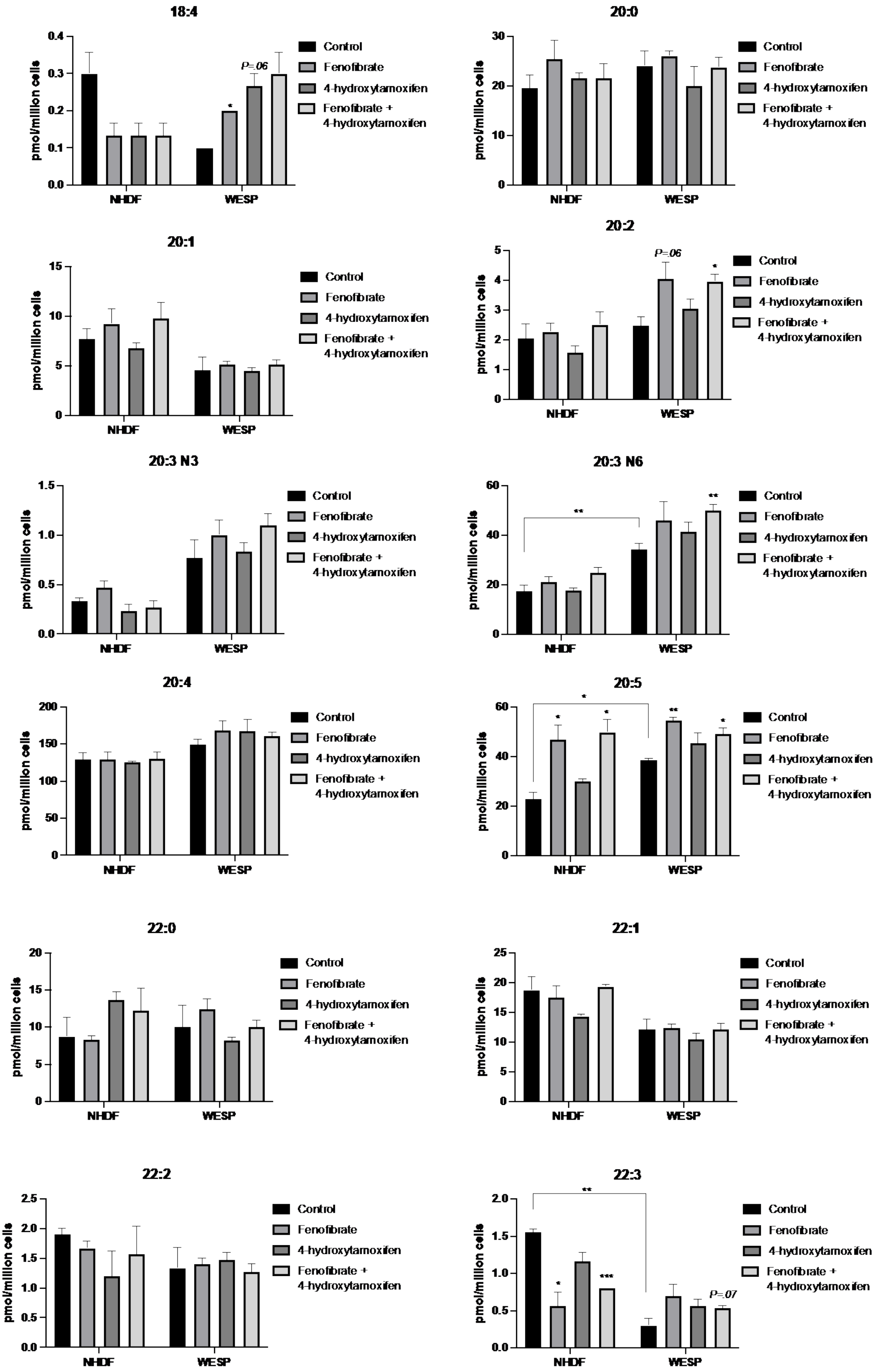

18:4
20:0
20:1
20:2
20:3 N3
20:3 N6
20:4
20:5
22:0
22:1
22:2
22:3
pmol/million cells
NHDF
WESP
Control
Fenofibrate
4-hydroxytamoxifen
Fenofibrate + 4-hydroxytamoxifen
P=.06
P=.07
*
**
***

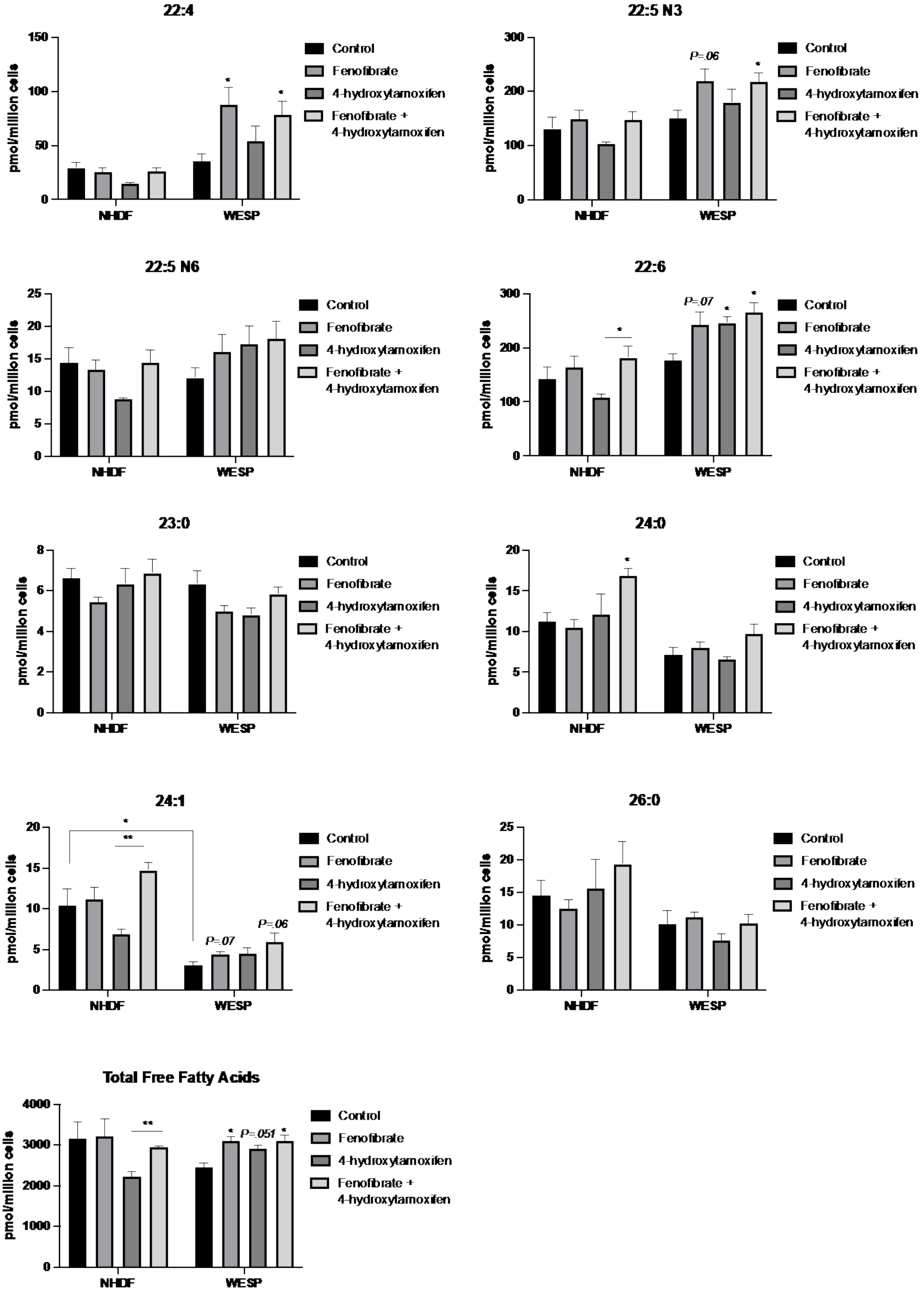

Additional file 3. Complete fatty acid profile of NHDF and WESP cells following treatment. Measurement of various species of free fatty acids after treatment with 25 μM fenofibrate, 0.5 μM 4-hydroxytamoxifen, or a combination of the two compounds for 24 hours. Data are shown as mean ± SEM (n=3). *$p < 0.05$; **$p < 0.01$; ***$p < 0.001$.
